# The Lamin-Like LITTLE NUCLEI 1 (LINC1) Regulates Pattern-Triggered Immunity and Jasmonic Acid Signaling

**DOI:** 10.3389/fpls.2019.01639

**Published:** 2020-01-09

**Authors:** Mai Jarad, Kiruthiga Mariappan, Marilia Almeida-Trapp, Michael Florian Mette, Axel Mithöfer, Naganand Rayapuram, Heribert Hirt

**Affiliations:** ^1^DARWIN21, Biological and Environmental Sciences and Engineering Division (BESE), King Abdullah University of Science and Technology (KAUST), Thuwal, Saudi Arabia; ^2^Research Group Plant Defense Physiology, Max Planck Institute for Chemical Ecology, Jena, Germany

**Keywords:** nuclear envelope, lamin, pathogen-associated molecular pattern, innate immunity, jasmonic acid, glucosinolate biosynthesis

## Abstract

Pathogen-associated molecular pattern (PAMP) recognition occurs by plasma membrane located receptors that induce among other processes nuclear gene expression. However, signaling to the nuclear compartment is restricted by the nuclear envelope and nuclear pore complexes. We show here that among the four *Arabidopsis* lamin homologs LITTLE NUCLEI/CROWDED NUCLEI (LINC/CRWN), LINC1 plays an important role in PTI and jasmonic acid (JA) signaling. We show that *linc1* knock out mutants affect PAMP-triggered MAPK activation and growth inhibition, but not reactive oxygen species or callose accumulation. We also demonstrate that *linc1* mutants are compromised in regulating PAMP-triggered pathogen-related genes, in particular encoding factors involved in JA signaling and responses. Expression of a number of JAZ domain proteins, the key JA-related transcription factor MYC2 as well as key MYB transcription factors and biosynthesis genes of both the indole and aliphatic glucosinolate pathways are changed in *linc1* mutants. Moreover, PAMP triggers JA and JA-Ile accumulation in *linc1* mutants, whereas salicylic acid levels are unchanged. Despite impairment in PAMP-triggered immunity, *linc1* mutants still show basal immunity towards *Pseudomonas syringae* DC3000 strains. High JA levels usually render plants resistant to necrotrophic pathogen. Thus, *linc1* mutants show enhanced resistance to *Botrytis cinerea* infection. In accordance with a general role of LINC1 in JA signaling, *linc1* mutants are hypersensitive to growth inhibition to external JA. In summary, our findings show that the lamin-like LINC1 protein plays a key role in JA signaling and regulation of PTI responses in *Arabidopsis*.

## Introduction

Plants are constantly under attack from potentially pathogenic microbes. The perception of a pathogen attack by the plant triggers the activation of immune signaling events and defense responses. Components of plant innate immunity have been categorized into two general lines of defense (Jones and Dangl, 2006), with pattern-triggered immunity (PTI) through the perception of pathogen-associated molecular patterns (PAMPs) by membrane bound pattern recognition receptors representing the first (Schwessinger and Ronald, 2012; Macho and Zipfel, 2014). Early defense responses include a rise in the intracellular calcium concentration (Liese and Romeis, 2013), the production of reactive oxygen species (ROS) (Torres et al., 2006), the activation of mitogen-activated protein kinase (MAPK) cascades and the expression of pathogen-related proteins (Nurnberger et al., 2004; Ahuja et al., 2012), and the biosynthesis of stress-related hormones such as salicylic acid (SA), jasmonic acid (JA), and ethylene (Grant and Jones, 2009; Tsuda et al., 2013). These early events after PAMP perception lead to defense responses mediated by transcriptional reprogramming (Mur et al., 2008), stomatal closure, production of antimicrobial compounds (Dixon, 2001), and the deposition of callose (Gomez-Gomez et al., 1999). The second tier of the plant defense system elicits a more robust and long-term response and is termed effector-triggered immunity. Effector-triggered immunity is prompted when effectors transferred by host-adapted pathogens in order to interrupt signaling steps of the PTI response are recognized by Resistance proteins of the host cells (Jones and Dangl, 2006; Nicaise et al., 2009; Collier and Moffett, 2009; Dodds and Rathjen, 2010; Tsuda and Katagiri, 2010; Hann et al., 2010; Spoel and Dong, 2012; Takken and Goverse, 2012).

JA not only regulates plant development, but also plays a pivotal role in local and systemic pathogen resistance responses. JA-Ile is the bio-active form of JA, it induces the formation of a receptor complex that leads to the ubiquitination and degradation of the JAZ (JASMONATE ZIM-DOMAIN) transcriptional repressors (Thines et al., 2007; Browse, 2009; Fonseca et al., 2009; Koo et al., 2009; Yan et al., 2016). In the absence of pathogen challenge, low levels of JA-Ile allow JAZ accumulation and repression of JAZ target genes (Pauwels and Goossens, 2011; Zhang et al., 2015). Upon pathogen-induced JA-Ile accumulation, JAZs interact with the F-box CORONATINE INSENSITIVE 1 (COI1), forming a co-receptor complex leading to JAZ degradation and transcriptional induction (Pauwels and Goossens, 2011).

Signal transduction induced by extracellular factors such as PAMPs ultimately results in modulation of gene expression in the nuclear compartment. However, access to the genomic DNA embedded in chromatin is restricted by the nuclear envelope and transport through nuclear pore complexes (NPCs). NPCs, which are embedded in the nuclear envelope, are surrounded by inner and outer nuclear membrane proteins. These NPCs include the SUN (Sad1p, UNC-84) and the membrane-spanning KASH (Klarsicht, ANC-1, Syne Homology domain) proteins (Poulet et al., 2017). A series of plant-specific WPP domain proteins play an analogous role to the metazoan KASH proteins including the anchoring of the RanGAP GTPase to the outer nuclear membrane (Xu et al., 2007; Zhao et al., 2008; Zhou et al., 2012). The protein complex that is formed by the KASH and SUN proteins is proposed to link the cytoskeleton to the nucleoskeleton. SUN1 and SUN2 interact with the LITTLE NUCLEI1 (LINC1)/CROWDED NUCLEUS1 (CRWN1) protein that lies at the periphery of the nucleoplasm, providing shape and size to the nucleus (Graumann, 2014). On the other hand, SUN proteins also interact with the WPP-INTERACTING PROTEIN (WIP1) protein that lies on the outer nuclear membrane (Zhou et al., 2012). WIP1 is closely related to WIT proteins, which link the nuclear envelope to actin filaments *via* the plant-specific KAKU1 myosin-like protein (Tamura et al., 2013).

In *Arabidopsis*, the *LITTLE NUCLEI/CROWDED NUCLEI* gene family has four members, *LINC1* to *LINC4*. While *linc1* and *linc4* single mutants are affected in nuclear size, nuclear shape, and chromatin organization, some double and triple mutants also show a dwarf phenotype (Dittmer et al., 2007; Wang et al., 2013). A recent study reported that LINC1 and LINC3 are involved in seed germination by regulating the degradation of ABA-INSENSITIVE5 (ABI5) protein (Zhao et al., 2016). Furthermore, LINC1 also interacts with the NAC transcription factor NTL9 and plays a role in suppressing the plant immune response towards the virulent pathogen *Pseudomonas syringae* pv. *maculicola* (*Psm*) ES4326 (Guo et al., 2017). Guo et al. also reported a role for pathogens and SA in inducing LINC1 degradation, which interacts with NTL9 and SNI1 to inhibit *PR1* transcription (Guo et al., 2017).

Here, we demonstrate that LINC1 plays a role in regulating PTI responses. By characterizing PTI in *linc1* knock out mutants, we show that LINC1 influences several components of the JA and glucosinolate (GS) biosynthetic and signaling pathways. The induction of the immune response in the *linc1* mutant plants is affected and leads to induction in JA accumulation and transcriptional reprogramming. Furthermore, both tryptophan-derived indole glucosinolate (IGS) and methionine-derived aliphatic glucosinolate (AGS) signaling pathways are affected in *linc1* mutants in PTI. The data reported in this study establish that LINC1 is a positive regulator of PTI responses and plays a role in JA signaling and GS biosynthesis.

## Results

### LINC1 Positively Regulates Early and Late PTI Responses

LINCs contain a tripartite structure with a central coiled coil domain as well as nuclear localization signals and are proposed to be the best candidates of lamins in *Arabidopsis* (Guo and Fang, 2014). They have been shown to participate in functions such as in regulating chromatin organization and nuclear shape and size that are controlled by nuclear lamins in organisms other than plants. *Arabidopsis* quadruple *linc* mutants are not viable (Dittmer et al., 2007; Sakamoto and Takagi, 2013; Wang et al., 2013), but mutations in both *LINC1* and *LINC4*, but not in *LINC2* and *LINC3*, can change nuclear size and the shape of differentiated cells (Dittmer et al., 2007; Dittmer and Richards, 2008). However, except for recent reports on the role of *LINC1* and *LINC3* in seed germination (Zhao et al., 2016), and enhanced disease phenotype in the *linc1 linc2* double mutant (Guo et al., 2017), the functions of LINC proteins have not been further characterized.

**Figure 1:**
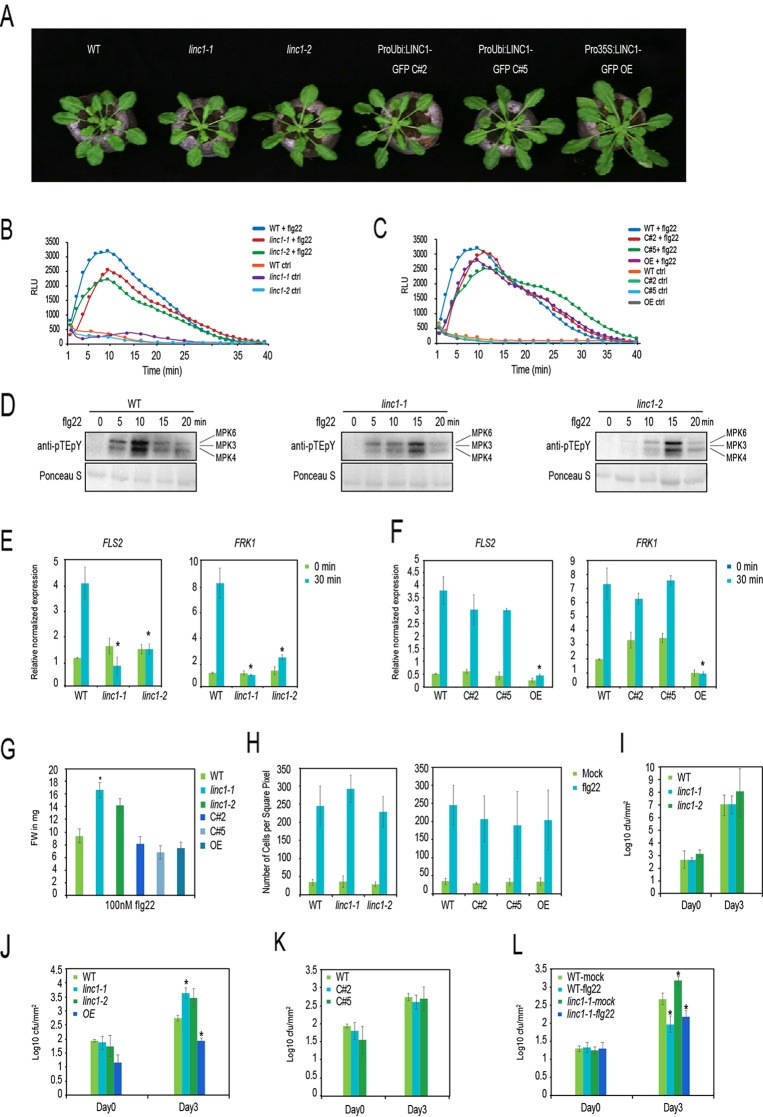
*LINC1* regulates early and late PTI responses. **(A)** Morphological phenotypes of 4-week old jiffy grown plants. **(B)** flg22-induced ROS burst over 40 min in leaf discs of 5-week old plants WT, *linc1-1*, *linc1-2* plants and **(C)** in WT, C#2, C#5, OE plants. **(D)** flg22-induced MAPK activation in WT, *linc1-1* and *linc1-2* plants. **(E**, **F)** FLS2 and FRK1 marker gene expression in 10-day old seedlings treated with 1 µM flg22 for 30 min of WT, linc1-1, linc1-2 plants and in WT, C#2, C#5, OE plants. **(G)** flg22-induced seedling growth inhibition of WT, *linc1-1*, *linc1-2*, C#2, C#5, and OE plants. **(H)** flg22-induced callose deposition in WT, linc1-1, linc1-2, C#2, C#5, and OE plants. **(I)** Bacterial population evaluated in 4-week old WT, *linc1-1*, *linc1-2* plants after 3 and 72 hpi with *Pst DC3000* at an OD 600 nm = 0.02. **(J)** Bacterial population evaluated in 4-week old WT, *linc1-1*, *linc1-2*, and OE plants after 3 and 72 hpi with *Pst hrcC*- at an OD 600nm = 0.02. **(K)** Bacterial population evaluated in 4-week old WT, C#2 and C#5 plants after 3 and 72 hpi with *Pst hrcC* at an OD 600 nm = 0.02. **(L)** Bacterial population evaluated in 4-week old WT and *linc1-1* plants pretreated with water or 1 1 µM flg22 for 24 hours and then challenged with Pst DC3000 at an OD 600 nm = 0.02 after 3 and 72 hpi. Flg22 priming of WT and linc1-1 plants with flg22 at 24 h before Pst hrcC- infection. The data are shown in **(E**–**L)** are means from three biological replicates with a two-way Anova. Asterisk indicates a significant difference with *P* < 0.05 when compared with the wild type.

To elucidate the specific role of *LINC1* in the PTI stress response, we investigated whether LINC1 is involved in flg22-induced PTI responses. We obtained T-DNA insertion lines *linc1-1* (Dittmer et al., 2007) and *linc1-2* (Sakamoto and Takagi, 2013) and confirmed by quantitative RT-PCR analysis (qRT-PCR) that both are knock out mutants (**Figures S1A, S1B**). Nevertheless, as reported before, both had no visible developmental or growth phenotype (**Figure 1A**). In order to generate complementation lines, we introduced a GFP-tagged *LINC1*-fusion construct under the control of a constitutive ubiquitin gene-derived promoter into the *linc1-1* mutant line by *Agrobacterium*-mediated transformation. Two of the obtained transgenic lines, *ProUbi: LINC1*-GFP C#2 and *ProUbi: LINC1*-GFP C#5, showed similar *LINC1* transcript levels as wild type plants (**Figure S2**) and exhibited no discernable growth or developmental phenotypes (**Figure 1A**). We also acquired a strong *LINC1* overexpression line, *Pro35S:LINC1-*GFP OE (Sakamoto and Takagi, 2013), this line showed 20-fold increased *LINC1* transcript levels and the plants were phenotypically slightly larger in size when compared to wild type (**Figures 1A** and **S1**).

We then proceeded to examine known early and late PTI responses in the *linc1* mutants. PAMP-induced ROS production is one of the well-documented early immune responses, therefore we measured ROS levels upon flg22 treatment in our selected lines. *Linc1-1* and *linc1-2* mutant plants showed slightly lower flg22-induced ROS levels, whereas the OE, C#2, and C#5 plants showed similar ROS levels as wild type plants (**Figures 1B**, **C**). Another well documented early PTI response is the activation of the immune MAPKs MPK3, MPK4, and MPK6 in response to flg22, which we checked by testing for activation-specific protein phosphorylation. *linc1-1* and *linc1-2* mutants displayed a delayed peak of activation of the MAPKs when compared to wild type (**Figure 1D**).

Furthermore, *linc1-1* and *linc1-2* mutants were completely compromised in the flg22-induced expression of the PTI marker genes *FLS2* and *FRK1* (**Figure 1E**). To confirm that the compromised *FLS2* and *FRK1* expression in these lines was due to the lack of functional *LINC1* we examined C#2 and C#5 lines which had been obtained by transforming the *linc1-1* mutant. Expression of *LINC1-GFP* restored flg22-mediated induction of *FLS2* and *FRK1* expression to normal levels (**Figure 1F**). We also examined the *LINC1 OE* line for *FLS2* and *FRK1* expression and found that it was compromised in flg22-mediated *FLS2* and *FRK1* induction (**Figure 1F**).

One of the later plant responses to PAMP perception is growth inhibition and increased callose deposition (Zhang et al., 2007). *Linc1-1* and *linc1-2* mutant plants exhibited resistance to growth inhibition by flg22 compared to wild type plants, while *LINC1 OE* and both complementation lines C#2 and C#5 exhibited normal growth-inhibition responses (**Figure 1G**). In addition, we measured callose accumulation in our mutant plants as well as in the OE line 24 h after flg22 treatment. However, no significant changes in callose accumulation levels were observed in any of the lines when compared to wild type plants (**Figure 1H**).

Finally, we tested if *LINC1* plays a role during PTI. We infected both *linc1-1* and *linc1-2* plants by spray-inoculation with the virulent bacterium *P. syringae* pv. *tomato* DC3000 (*Pst* DC3000), and the non-virulent *P. syringae* pv. *tomato* DC3000 *hrcC*^−^ (*Pst hrcC*^−^). The *Pst hrcC*^−^ strain is characterized by attenuated virulence due to a lack of a functional type III-secretion system and therefore can be considered as a PTI-specific strain. We observed no difference between bacterial levels within leaf tissue in wild type, *linc1-1*, and *linc1-2* plants after spray-inoculation with *Pst* DC3000 at 3 or 72 h post-inoculation (hpi) (**Figure 1I**), indicating that *LINC1* does not play an essential role in basal resistance. However, a reduction in resistance was evident in *linc1-1* and *linc1-2* after 72 h post-inoculation with *Pst hrcC*^−^ (**Figure 1J**). Since no differences in bacterial levels were observed at 3 h, we could exclude an effect at the level of bacterial access through stomata. The enhanced susceptibility to infection in the *LINC1* knock out mutants was largely restored to wild type levels in the complementation lines C#2 and C#5 (**Figure 1K**). To assess the specificity of the enhanced plant resistance detected in *linc1* mutants, we tested whether a similar phenotype could be observed in T-DNA knockout mutant lines of other members of the *LINC* gene family (Wang et al., 2013). To test whether flg22-induced priming is affected in linc1, we pretreated wild type and linc1-1 plants with flg22 24 h before Pst hrcC- infection. Wt and linc1-1 plants showed similar priming, indicating that linc1-1 is not compromised in flg22 priming (**Figure 1L**). We did not find significant changes in response to *Pst hrcC*^−^ infection in *linc2*, *linc3*, *linc4*, *linc1/linc4*, or *linc2/linc3* plants at 72 hpi (**Figure S3**). Interestingly, although we did not see major effects of the OE lines in PTI markers, these plants showed lower infection levels than in wild type plants. Moreover, since Guo et al. reported that among the single and double *linc* mutant lines tested, they observed a slightly enhanced pathogen resistance phenotype to the virulent *Psm* ES4326 pathogen in the single *linc1-1* mutant, we decided to investigate whether a similar phenotype could be observed in the *linc1* mutants plants used in this study. We observed no significant changes in response to *Psm* ES4326 pathogen infection in *linc1-1*, *linc1-2*, *OE*, *C#2*, and *C#5* plants after 72 hpi (**Figure S4**).

Taken together, our data demonstrate that the disease resistance to *Pst hrcC*^−^ is strongly affected in *linc1* mutant plants. Moreover, these results also indicate that *LINC1* plays a significant role in the regulation of PAMP-induced MAPK activation and defense gene expression.

### *LINC1* Regulates PAMP-Triggered Gene Expression

**Figure 2:**
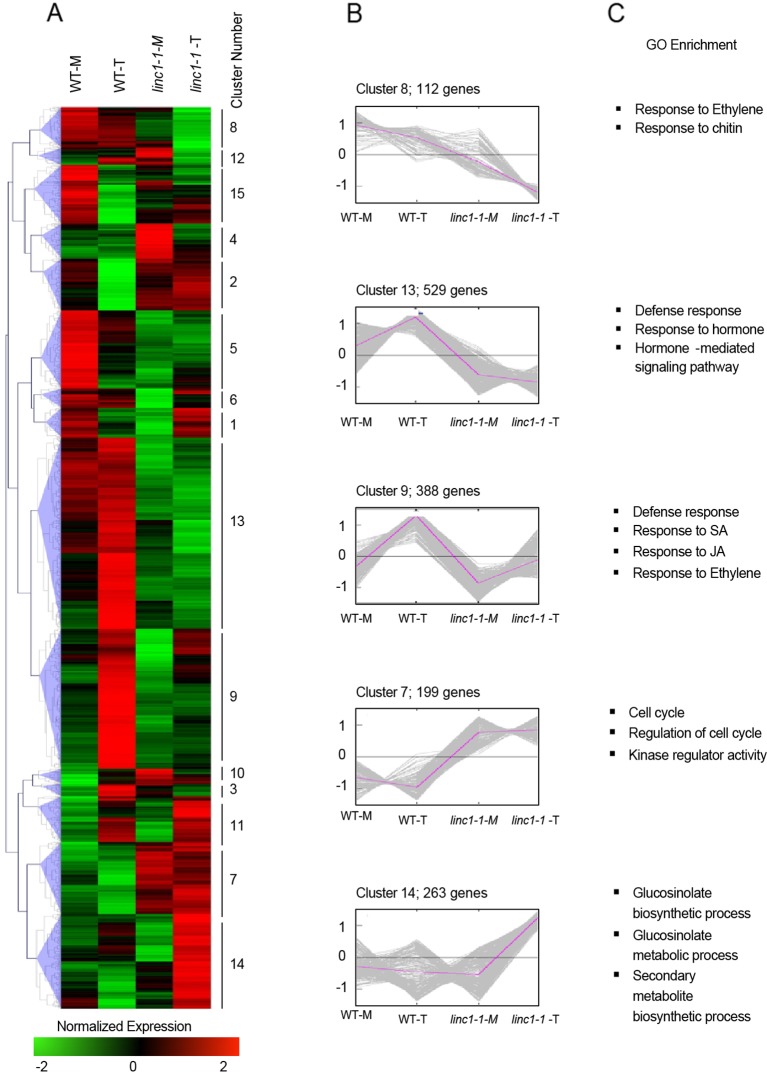
*LINC1* globally regulates expression of *Pst hrcC*^−^ induced genes. **(A)** Heat map of mock and *Pst hrcC*^−^ induced genes in wild type and *linc1-1* plants. The original FPKM values were subjected to data adjustment by normalized genes/rows and hierarchical clustering was generated with the average linkage method using MeV4.0. Red color indicates relatively high expression, and green indicates relatively low expression. **(B)** Expression pattern of the gene clusters defined in panel **A**. **(C)** Enrichment of genes with GO terms of significantly enriched gene families. The fold enrichment was calculated based on the frequency of genes annotated to the term compared with their frequency in the genome.

In order to identify genes dependent on *LINC1* in their regulation after *Pst hrcC*^−^ treatment, we performed a global transcriptome analysis on samples from three independent repeats of 14 day old seedlings of wild type and *linc1-1* mutant plants (**Figure 2**). The plants were harvested at 24 hpi of *Pst hrcC*^−^. Of the 24,911 transcripts that were represented in the data, 1618 genes showed differential expression (fold change > 1.5, p value < 0.05). Comparing non-treated *linc1-1* to wild type plants, we found 420 upregulated and 912 down regulated genes. Comparing mock treated to *Pst hrcC*^−^ treated samples, we found 786 upregulated and 832 downregulated genes in wild type compared to 865 upregulated and 456 down regulated genes in *linc1-1* (fold change > 1.5, P value < 0.05). When we compared the differentially regulated genes (DEGs) in *Pst hrcC*^−^ treated *linc1-1* and *Pst hrcC*^−^ treated wild type plants, we found that 55% of *linc1-1 Pst hrcC*^−^ DEGs are downregulated (585 genes), whereas 45% are up-regulated (385 genes) (**Figure S5A**).

GO analysis of genes down regulated in *linc1-1* inoculated with *Pst hrcC*^−^ in comparison to wild type inoculated with *Pst hrcC*^−^ showed a clear enrichment in genes involved in defense response, response to hormones, and hormone mediated signaling pathways (**Figure S5B**). In contrast, up-regulated genes were enriched in cell cycle-, GS biosynthesis-, and metabolism-related functions (**Figure S5C**).

To gain a deeper understanding of the role of *LINC1*, we carried out hierarchical clustering of *Pst hrcC*^−^ induced genes in *linc1-1*, which were then separated into 15 clusters and examined by GO enrichment (**Figure 2A**). Clusters 9, 13, and 14 contained the largest numbers of DEGs with 388, 529, and 263 genes, respectively, revealing that in *linc1-1* mutants, immune-related genes are repressed when compared to the situation of *Pst hrcC*^−^ induced genes in wild type (**Figure 2B**). A number of GO categories that are all associated with defense processes, including systemic acquired resistance, and the key defense hormones ethylene (ET), SA, and JA, were significantly compromised in *linc1-1* mutant plants upon *Pst hrcC*^−^ treatment. Interestingly, a strong upregulation of secondary metabolite and GS biosynthesis and metabolism was also observed in *Pst hrcC*^−^ challenged *linc1-1* mutant plants (**Figure 2C**).

### *LINC1* Modulates Defense and Hormone Related Genes

**Figure 3:**
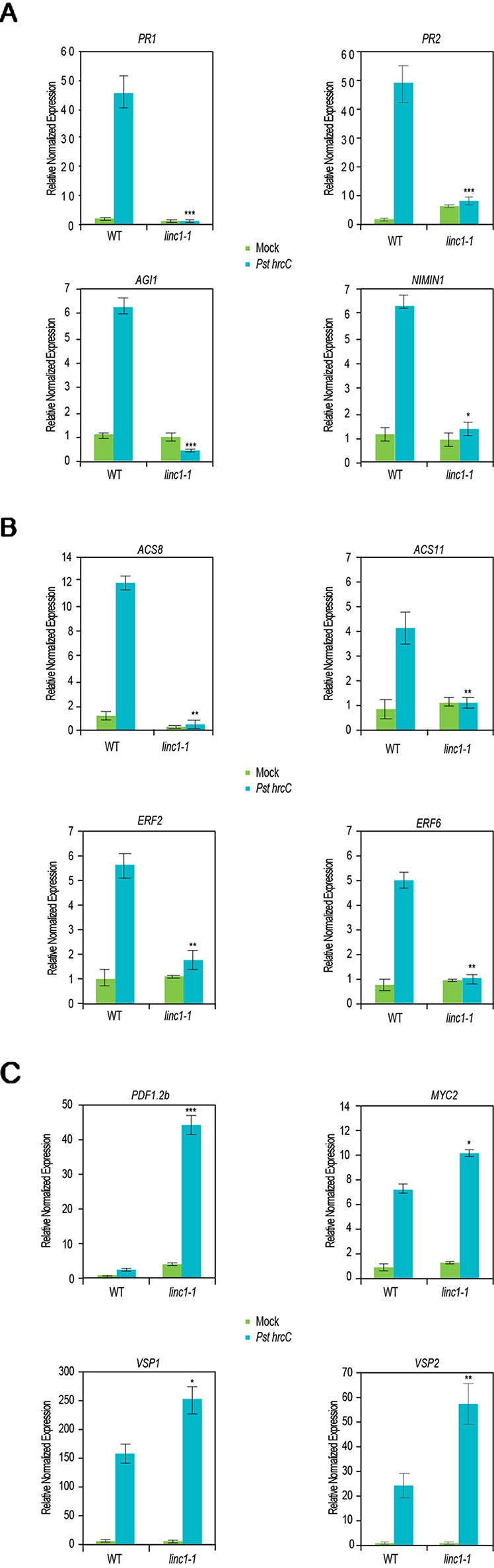
*LINC1* modulates defense and hormone related genes. 14-d-old seedlings were treated with Pst hrcC- for 24 h and harvested later to isolate RNA and cDNA preparation. **(A)** Quantitative RT-PCR (qRT-PCR) analysis of salicylic acid (SA)-related genes regulated by *LINC1*. **(B)** qRT-PCR analysis of ethylene (ET)-related genes regulated by *LINC1*. **(C)** qRT-PCR analysis of jasmonic acid (JA)-related genes regulated by *LINC1*. Gene expression was normalized to internal control *UBQ10* and actin. The data are shown in **(A**–**C)** are means from three biological replicates. Statistical significance was analyzed by two-way ANOVA, asterisk indicate significant differences compared to Wild Type, *p ≤ 0.05, **p ≤ 0.01, ***p ≤ 0.001.

To further examine the mechanisms by which *LINC1* regulates hormone-related genes, we performed qRT-PCR for SA-, ET-, and JA-related genes upon *Pst hrcC*^−^ treatment. QRT-PCR confirmed the RNA-seq results indicating a repression in the expression of the SA-related genes *PR1*, *PR2*, *AIG1*, and *NIMIN1* in the *Pst hrcC*^−^ treated *linc1-1* mutant when compared to *Pst hrcC*^−^ treated wild type plants (**Figure 3A**). Similarly, the ET-related genes *ACS8*, *ACS11*, *ERF2*, and *ERF6* also exhibited repressed gene expression in the *Pst hrcC*^−^ treated *linc1-1* mutant when compared to *Pst hrcC*^−^ treated wild type plants (**Figure 3B**). On the other hand, the JA-related genes *MYC2*, *PDF1.2b*, *VSP1*, and *VSP2* showed a marked increase in the expression in both the mock and *Pst hrcC*^−^ treated plants when compared with wild type plants (**Figure 3C**). These results confirm the transcriptome data and point to an important role for *LINC1* in modulating the expression of defense-related hormone genes. We were able to see recovery of the expression of all of these genes to wild type levels in the C#2, C#5, and OE lines (**Figures S6**, **S7**, **S8**).

#### Effect of *LINC1* on JA Accumulation and Signal Transduction

**Figure 4:**
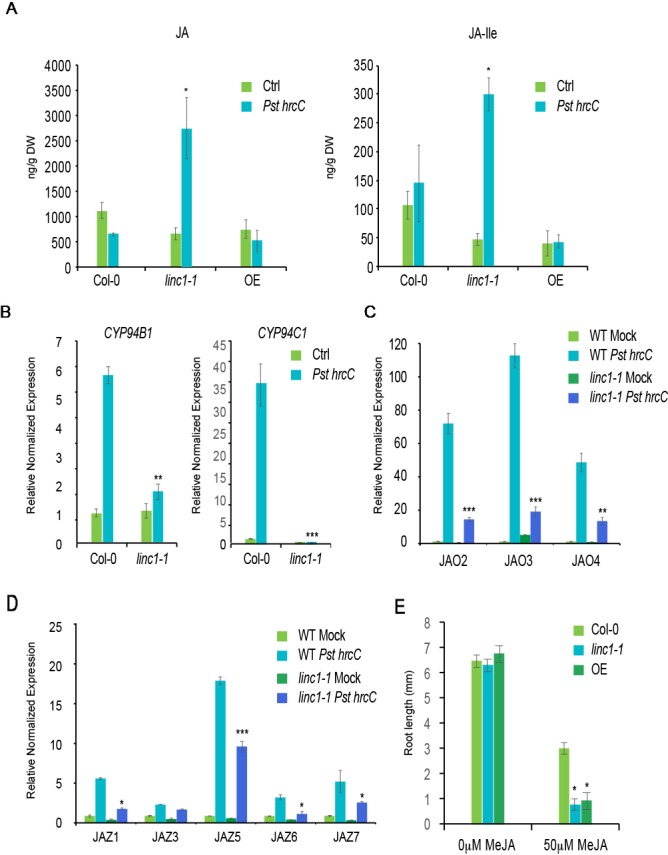
*LINC1* modulates JA accumulation and signaling. **(A)** JA and JA-Ile levels in wild-type, *linc1-1* and *OE* plants. Leaves of 3–4 weeks-old wild-type and *linc1-1* mutant were infiltrated with *Pst hrcC*- (OD 600 nm = 0.2). **(B)** 14-d-old seedlings were treated with *Pst hrcC*- for 24 h and harvested later to isolate RNA and cDNA preparation. qRT-PCR analysis of *CYP94B1* and *CYP94C1* genes regulated by *LINC1*. **(C)** qRT-PCR analysis of *JAO2*, *JAO3* and *JAO4* genes regulated by *LINC1*. **(D)** qRT-PCR analysis of JAZ1,3,5,6 and 7 genes regulated by *LINC1*. Gene expression was normalized to internal control UBQ10 and actin. **(E)** Both *linc1-1* and *OE* plants show increased sensitivity to MeJA compared to wild type plants. Values are averages ± SE of three biological replicates consisting of pools of 12 seedlings. The data are shown in **(A**–**E)** are means from three biological replicates. Statistical significance was analyzed by two-way ANOVA, asterisk indicate significant differences compared to Wild Type, *p ≤ 0.05, **p ≤ 0.01, ***p ≤ 0.001.

Both the transcriptome data as well as the qRT-PCR results indicated that a substantial number of JA-related genes are significantly affected in the *linc1-1* mutant. Since these changes in JA-related gene expression could affect endogenous JA and JA-Ile levels, we quantified their levels in the *LINC1* knock out and the OE lines. We found a significant increase in JA and JA-Ile levels in the *linc1-1* mutant compared with wild type plants following inoculation with *Pst hrcC*^−^ at 24 hpi, while JA-Ile levels exhibited a reduction in the OE line (**Figure 4A**). High JA levels are known to boost resistance to necrotrophic pathogens. Therefore, we examined the *linc1* mutant plants as well as the OE line for sensitivity to the necrotrophic fungal pathogen *Botrytis cinerea*. The *linc1* mutants were more resistant against *B. cinerea*, while the OE line exhibited a wild type-like response (**Figure S9A** and **B**). Furthermore, we found no changes in SA levels in the *linc1-1* mutant and the OE line compared with wild type plants following inoculation with *Pst hrcC*^−^ at 24 hpi (**Figure S9C**).

Recent studies highlighted a transcriptionally activated JA-Ile catabolic pathway, which is mediated through three endoplasmic reticulum (ER)-localized cytochrome P450 oxidases, CYP94B1, CYP94B3, and CYP94C1, that induce JA break-down (Heitz et al., 2012; Koo et al., 2014). From our transcriptome data, we found that the expression of CYP94B1 and CYP94C1 was significantly inhibited following *Pst hrcC*^−^ treatment. In order to confirm this observation, we performed qRT-PCR analysis on CYP94B1 and CYP94C1 expression levels. *Pst hrcC*^−^ induced expression of CYP94B1 and CYP94C1 was compromised in the *linc1-1* mutant when compared to WT (**Figure 4B**). In contrast, recovery of the induction of these two genes to wild type levels was observed in C#2, C#3, and OE lines (**Figure S10**).

A newly characterized set of enzymes termed JAOs (JASMONIC ACID OXIDASES) encompassing four members, JAO1, JAO2, JAO3, and JAO4, has been demonstrated to inactivate JA by hydroxylation (Smirnova et al., 2017). The expression levels of JAO2, JAO3, and JAO4 were affected in our transcriptome analysis. qRT-PCR analysis confirmed that *Pst hrcC*^−^ induced expression of *JAO2*, *JAO3*, and *JAO4* is significantly compromised in *linc1-1* mutant plants when compared to wild type (**Figure 4C**). We also examined expression in the C#2, C#5, and OE lines and found that their induction levels are comparable to wild type plants (**Figure S11**).

To further validate the accumulation of JA and JA-Ile in the linc1-1 mutant on the observed gene expression pattern, we used qRT-PCR to examine the expression levels of several *JAZ* genes with a known role in repressing JA signaling. We found that *JAZ1*, *JAZ3*, *JAZ5*, *JAZ6*, and *JAZ7* are all significantly compromised in *Pst hrcC*^−^ induction in *linc1-1* mutants (**Figure 4D**), but expression of these *JAZ* genes was found at comparable levels to wild-type in C#2, C#5, and OE lines (**Figure S12**).

The increased expression of several JA responsive genes and the inhibition of several JA-repressor genes suggested that the response to JA might be altered in *LINC1* mutant plants. Since MeJA inhibition of root growth is a classical JA assay, we tested the effect of MeJA treatment on the roots of the linc1-1 mutant and *LINC1* OE line. We observed that following MeJA treatment of the *linc1-1* mutant, root growth was much more inhibited than in wild type plants (**Figure 4E**). Surprisingly, *LINC1* OE also resulted in enhanced root growth inhibition, suggesting that appropriate LINC1 protein levels are necessary for JA signaling. Taken together, our expression analysis and measurement of JA and JA-Ile levels, indicate that *linc1* is a positive regulator of JA biosynthesis and signaling.

### *LINC1* Regulates GS Biosynthetic and Metabolic Pathways

Another transcriptome segment revealed that transcript levels of several members of the GS biosynthetic pathways are enhanced in *linc1* mutants upon *Pst hrcC*^−^ infection (**Figure 2**, Cluster 14). GS are a large group of secondary metabolites in *Brassicaceae* that are involved in plant defense against insects as well as pathogens (Brader et al., 2006; Hopkins et al., 2009). GS are amino acid-derived compounds and comprise the tryptophan-derived IGS and methionine-derived AGS. Several R2R3-MYB transcription factors initiate the expression of GS biosynthetic genes. *MYB28*, *MYB29*, and *MYB76* drive AGS biosynthesis (Celenza et al., 2005; Gigolashvili et al., 2007; Sonderby et al., 2007; Gigolashvili et al., 2008; Sonderby et al., 2010a; Li et al., 2013), while *MYB34*, *MYB51*, and *MYB122* transcriptionally regulate IGS biosynthesis genes (Frerigmann and Gigolashvili, 2014).

**Figure 5:**
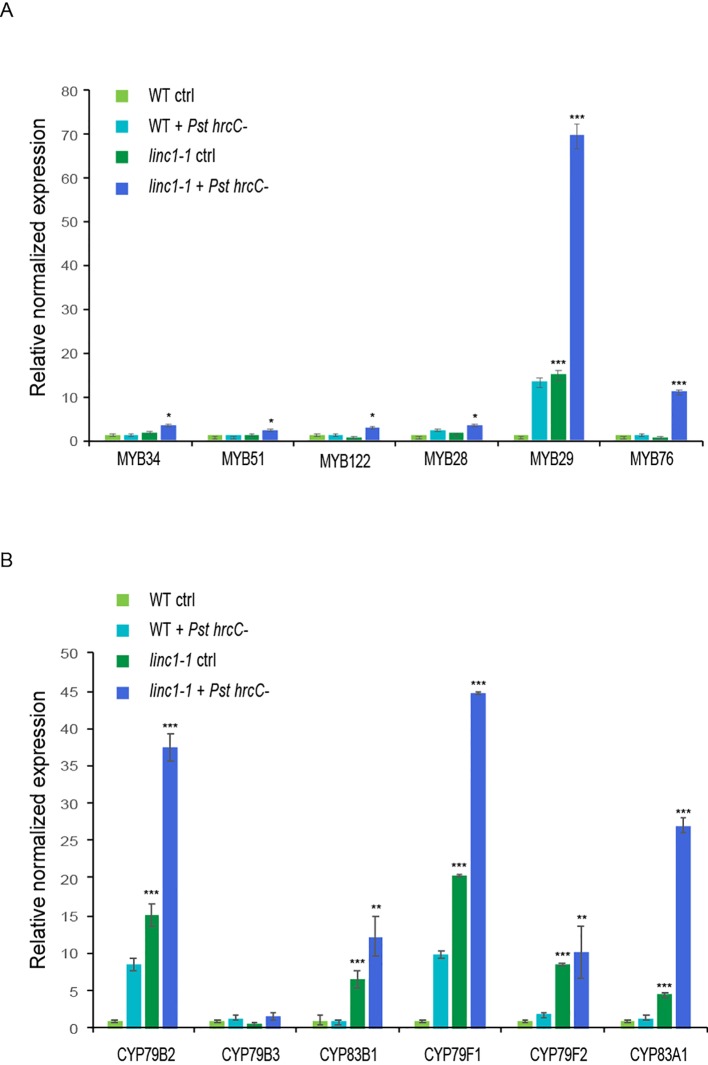
*LINC1* modulates the glucosinolate biosynthesis pathway. 14-d-old seedlings were treated with Pst hrcC- for 24 h and harvested later to isolate RNA and cDNA preparation. **(A)** qRT-PCR analysis of expression levels of transcription factor genes involved in the biosynthetic pathway of indolic and aliphatic glucosinolates. **(B)** qRT-PCR analysis of expression levels of biosynthetic genes involved in the biosynthetic pathway of indolic and aliphatic glucosinolates. Gene expression was normalized to internal control *UBQ10* and actin. The data are shown in **(A**, **B)** are means from three biological replicates. Statistical significance was analyzed by two-way ANOVA, asterisk indicate significant differences compared to Wild Type, *p ≤ 0.05, **p ≤ 0.01, ***p ≤ 0.001.

IGS and AGS synthesis is mostly JA-induced (Sonderby et al., 2010b). The increased accumulation of JA and JA-Ile levels in *linc1-1* mutants led us to explore JA-induced GS synthesis. Before and after *Pst hrcC*^−^ treatment, the expression *of MYB28*, *MYB29*, *MYB76*, *MYB34*, *MYB51*, and *MYB122*, was significantly upregulated in *linc1-1* mutants when compared with wild type plants (**Figure 5A**). Enhanced *MYB* transcription factor mRNA levels also correlated with upregulated expression of key biosynthetic genes, such as *CYP79B2*, *CYP79B3*, and *CYP83B1* which are related to IGS synthesis, and *CYP79F1*, *CYP79F2*, and *CYP83A1*, which are involved in AGS biosynthesis (**Figure 5B**). The expression of GS genes was also assessed in our C#2, C#5, and OE lines, showing comparable levels to wild type (**Figures S13**, **S14**). These results demonstrate that the expression of all the tested IGS and AGS biosynthetic genes is affected in *linc1-1* mutants with and without *Pst hrcC*^−^ treatment.

## Discussion

The *Arabidopsis* genome contains four *LINC* genes which have been extensively documented regarding their roles in regulating nuclear structure, chromatin organization, and nuclear size (Dittmer et al., 2007; Sakamoto and Takagi, 2013; Wang et al., 2013; Guo and Fang, 2014; Grob et al., 2014). Recent work addressed the role of the *LINC* gene family in biological processes such as seed germination (Zhao et al., 2016) and plant immunity (Zhao et al., 2016; Guo et al., 2017; Choi et al., 2019). In the latter study, Guo et al. reported that among the single and double *linc* mutant lines tested, they observed a slightly enhanced pathogen resistance phenotype in the single *linc1-1* mutant against infection by the virulent *Psm* ES4326 pathogen, but a much stronger resistance phenotype in the *linc1 linc2* double mutant. Choi et al. (2019) recently also investigated the role of *LINC* genes in immunity. Upon *Pst* DC3000 infection, they found no difference in *linc1* and *linc4* single mutants but enhanced resistance in *linc1/2* and *linc1/4* double mutants. Guo et al. (2017) were able to observe lower SA levels in the *linc1 linc2* double mutant than in wild-type plants after inoculation with *Psm* ES4326, whereas Choi et al. (2019) found elevated SA levels only in *linc1/2* double mutants. In the *linc1/2* and *linc1/4* double mutants, the same authors reported enhanced levels of cell death and *SID2*, *PR1*, *2* and *5* mRNA amounts (Choi et al., 2019). These observations strongly indicate that members of the *LINC* gene family play a significant role in the plant immune response that necessitates further examination.

We examined if there is a specialized role for the *LINC* genes in the PTI response using inoculation with the PTI specific bacterial strain *Pst hrcC*^−^ as a model system. Out of four single and two double mutant *linc* lines examined, only *linc1* single mutants exhibited enhanced susceptibility to *Pst hrcC*^−^ infection. Additionally, our data further show that *linc1* knock out mutants are compromised in the PAMP-induced activation of MPK3, MPK4, and MPK6 as well as the expression of PTI marker genes. These results corroborate the enhanced susceptibility phenotype of *linc1* mutants to infection by non-virulent *Pst hrcC*^−^ indicating that *LINC1* plays a role in the transcriptional regulation of defense-related genes and is a positive regulator of PTI. We did not observe any changes in SA levels upon *Pst hrcC*^−^ infection, but significant changes in JA and JA-Ile levels and observed that *linc1* mutants show enhanced resistance to the nectrotrophic fungal pathogen *B. cinerea*. Since the single *linc1* mutants already show a role for *LINC1* in JA-related immune responses, we believe that this is the true function of LINC1, whereas double mutant combinations of several pathways might generate synthetic phenotypes.

Phytohormones play a key role in plant disease resistance, whereby pathogen infection-induced hormone level changes can result in pronounced effects on defense gene expression (Durrant and Dong, 2004). This prompted us to examine the global gene expression profile of a *linc1-1* knock out mutant in response to *Pst hrcC*^−^ infection. The data suggest that *LINC1* controls defense and hormone signaling genes in response to *Pst hrcC*^−^ infection. Furthermore, in response to infection, SA- and ET-related genes become significantly suppressed while JA response genes are strongly induced. These changes in the expression levels of hormone and immune related genes in response to *Pst hrcC*^−^ infection further support a pivotal role for *LINC1* in regulating PTI.

The substantial increase in the expression levels of the JA response genes in *linc1* mutant plants might result in an increase in endogenous JA and JA-Ile levels. Indeed, *linc1* knock out plants showed enhanced, whereas *LINC1* OE resulted in reduced JA and JA-Ile levels. Furthermore, our analysis of the *LINC1* KO and OE lines revealed no changes in SA levels when compared to wild-type after inoculation with *Pst hrcC*^−^. The increased JA levels together with the unchanged SA levels could explain the enhanced susceptibility of *linc1* mutant plants to *Pst hrcC*^−^ infection, while at the same time exhibiting a resistant phenotype toward *B. cinerea*.

Key components in JA signaling are the JAZ proteins, which repress the activity of transcription factors in the absence of JA-Ile. Upon JA-Ile perception, JAZs dissociate from their TFs to form co-receptor complexes with COI1 before their subsequent degradation. The JAZ-released TFs can then activate JA-responsive genes and related defense (Thines et al., 2007; Pauwels and Goossens, 2011). We therefore investigated whether the observed increased JA accumulation leads to changes in the expression of JAZ repressors. Compared to wild type, we detected reduced expression of *JAZ1*, *JAZ3*, *JAZ5*, *JAZ6*, and *JAZ7* in *Pst hrcC*^−^-untreated and treated *LINC1* KO plants. To further investigate the effect of an increased expression of several JA-responsive genes along with the decreased expression of several JA-repressor genes on JA responses in the *LINC1* lines, we performed a MeJA root growth inhibition assays. The inhibitory effect of MeJA on root growth was more pronounced in *LINC1* KO and OE plants. This result supports the idea that LINC1 protein levels might also control JA signaling in a developmental context, but needs further studies.

**Figure 6:**
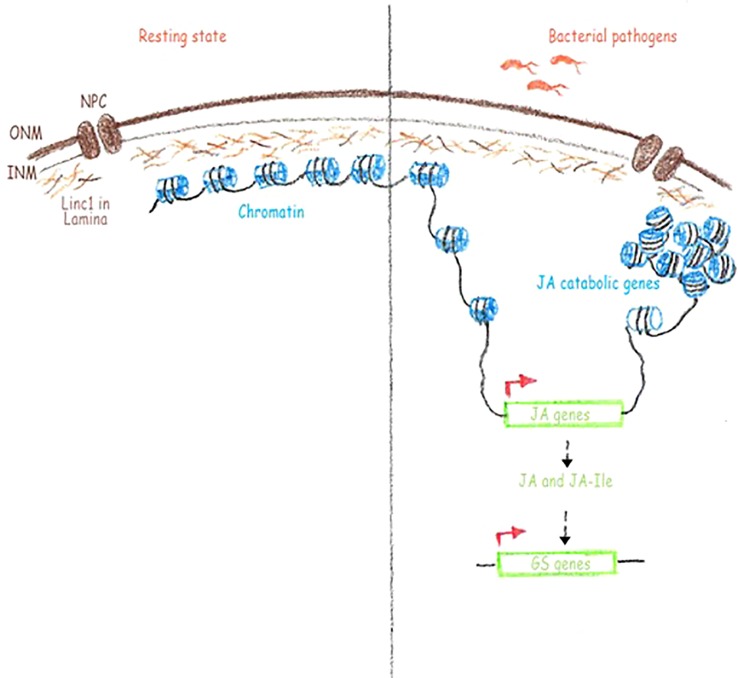
Hypothetical working model for the role of LINC1 in plant immunity. Linc1 is present in nuclear lamina and acts as a sort of scaffold to hold the chromatin. When necessary, such as during a pathogen infection, Linc1 facilitates the open conformation of the genes required for defense, such as the JA biosynthetic and signaling genes and closed conformation of certain other genes such as the JA catabolic genes resulting the accumulation of JA and JA-Ile. The increased amounts of JA and JA-Ile inturn switch on the expression of glucosinolate genes. ONM, outer nuclear membrane; INM, inner nuclear membrane; NPC, nuclear pore complex; JA, jasmonic acid; JA-Ile, jasmonic acid- isoleucine; Gs, glucosinate genes.

GS as well as their hydrolysis products have been demonstrated to play a role in the resistance to biotic stresses (Brader et al., 2006; Schlaeppi et al., 2010). Characterization of the components of the GS biosynthetic pathway have been carried out and both transcription factors controlling GS-related genes as well as core biosynthetic genes were identified (Halkier and Gershenzon, 2006; Brader et al., 2006). Positive regulation of several GS transcription factors and biosynthetic genes has been shown to be JA driven (Doughty et al., 1995; Brader et al., 2001; Mikkelsen et al., 2003). The increased JA and JA-Ile levels in *linc1* mutant plants prompted us to examine if there is an effect on the GS biosynthetic pathways. We found that the expression of *MYB* transcription factors involved in regulation of tryptophan-derived IGS and methionine-derived AGS biosynthesis, including *MYB28*, *MYB29*, *MYB76*, *MYB34*, *MYB51*, and *MYB122*, was significantly upregulated in both *Pst hrcC*^−^ treated and untreated *linc1* plants when compared with wild-type. Examination of the expression pattern of several IGS and AGS biosynthetic genes, such as *CYP79B2*, *CYP79B3*, *CYP83B1, CYP79F1*, *CYP79F2*, and *CYP83A1* under *Pst hrcC*^−^ treated and untreated conditions reveled that they were also highly induced in *linc1* mutant plants. In summary, the identification of a function of *LINC1* in both JA signaling and JA-Ile accumulation reveals a new role for this protein in the regulation of JA-related processes including immunity (**Figure 6**).

## Materials and Methods

### Plant Material and Growth Conditions

All *Arabidopsis thaliana* lines used in this study were in the Columbia-0 (Col-0) background which was used as wild-type plant. The following mutants and transgenic lines were used: *linc1-1* (SALK_025347; Dittmer et al., 2007; Wang et al., 2013), and *linc1-2* (SALK_016800; Sakamoto and Takagi, 2013). T-DNA insertions were confirmed by PCR using a specific primer of the T-DNA border (LBb1.3 primer for SALK lines: 5′-ATTTTGCCGATTTCGGAAC-3′) and gene-specific primers for newly described T-DNA lines are provided in **Table S1**. *Arabidopsis* plants were grown in Jiffy pots with a photoperiod of 16/8 h and at 22°C and 60% humidity, or on plates containing ½ Murashige-Skoog (MS) medium with a photoperiod of 16/8 h at 22°C. The *Nicotiana benthamiana* plants were soil-grown under a photoperiod of 16/8 h and at 28°C for 4 weeks and used for Agrobacterium infiltration for transient expression studies.

### Cloning and Transgenic Lines

The coding sequence of LINC1 was commercially synthesized from GenScript and cloned into the Gateway entry vector pENTR-D/Topo (Invitrogen). The gene was then shuttled using Gateway LR Clonase reaction (Invitrogen) into pDEST-His-MBP (Addgene) for recombinant protein expression and into pUBN (Addgene) for subcellular localization and generation of stable transgenic lines.

### *Pseudomonas* Infection Studies

Bacterial strains used in this study were *P. syringae* pv. *tomato* DC3000 (*Pst* DC3000), *P. syringae* pv. *tomato* DC3000 *hrcC (Pst hrcC*^−^*)*, and *P. syringae* pv. *maculicola* ES4326 (*Psm* ES4326). The strains were grown on NYGA media (5 g/L bactopeptone, 3 g/L yeast extract, 20 ml/L glycerol, and 15 g/L agar) containing rifampicin (50 mg/l) at 28°C for 48 h. For bacterial enumeration assays, plants were sprayed with the strains (inoculum: OD600 nm = 0.2 for *Pst* DC3000 and *Pst hrcC*^−^ and OD600 nm = 0.002 for *Psm* ES4326), suspended in 10 mM MgCl_2_ containing 0.001% (v/v) Silwet L-77 (Bio world). Sprayed plants were covered with a transparent plastic lid for the remaining time of the experiment. For bacterial titers, leaf discs from three different leaves per plant were harvested and washed, and then bacteria were extracted using 10 mM MgCl2 containing 0.04% (v/v) Silwet L-77. Quantification was done by plating appropriate dilutions on LB agar media containing rifampicin (50 mg/l) and incubated at 28°C for 2 days, after which the bacterial colonies were counted.

### *B. cinerea* Infection Studies

Inoculation with *B. cinerea* (**strain: B05.10)** for the microarray experiments was conducted on 4-week-old plants by placing a 5 μl droplet of a spore suspension (5*10^6^ spores/ml) on each rosette leaf (three fully expanded leaves per plant). Inoculated plants were covered with a transparent plastic dome to maintain high humidity and returned to the growth chamber for 72 h. For each biological replicate, inoculated leaves from three different plants were harvested and pictures were taken for lesion quantification.

### PAMP and Hormone Treatments

For PAMP treatments, flg22 synthesized peptide (GenScript Inc.), was used at 1 μM concentration. 50 μM of MeJA (Sigma Aldrich) was used for the root growth inhibition assay. Water treatment was used as mock treatment.

### Protein Extraction, SDS-PAGE, and Western Blot

Total protein was extracted from frozen leaf tissue, subjected to SDS-PAGE, transferred to a polyvinylidene fluoride membrane (Millipore), and used for immunodetection as described (Meskiene *et al*., 2003). Anti-pTpY antibody (Cell Signaling) was used to detect the activation loop conserved in the MAPKs. Antigen–antibody complexes were detected with horseradish peroxidase-conjugated anti-rabbit secondary antibody (Sigma-Aldrich), proteins were then detected using ECL Select™ Western Blotting Detection Reagent (GE Healthcare Amersham™) and the ChemiDoc MP imaging system (BioRad). Equal protein transfer was monitored by staining membranes with Ponceau S (Sigma-Aldrich) for 5 min.

### PAMP-Induced MAPK Activation

PAMP-induced MAPK activation assays were performed on 2-week-old seedlings grown in liquid medium. Seedlings were then elicited with 1 μM flg22 for 0, 5, 10, 15, and 30 min and then collected and frozen in liquid nitrogen. MAPK activation-specific phosphorylation was monitored by Western blot analysis as described with Phospho-p44/42 MAPK (Erk1/2) (Thr202/Tyr204) (D13.14.4E) XP rabbit monoclonal antibody (#4370, Cell Signaling) specific to dual phosphorylation of the activation loop of MAPK (pTEpY).

### PAMP-Induced Oxidative Burst

Twelve leaf discs (4 mm in diameter) of four 3–4 week old plants per genotype were collected. Each leaf disc was floated adaxial-side-up in an individual well of a 96-well microtiter plate (Costar, Fisher Scientific) containing 150 μl distilled H_2_O and then incubated overnight at 22°C in continuous light to reduce the immediate wounding response. Immediately prior to elicitation, the incubating medium was carefully removed from each well avoiding any tissue damage or desiccation. Using a multichannel pipet, then 100 μl of the elicitation solution [10 mg/ml horseradish peroxidase (HRP, Sigma-Aldrich), 0.2 μM Luminol (Sigma-Aldrich), 1 μl flg22 (1mM)] was quickly added to each well containing leaf disc for the treated samples and without the 5 μl flg22 for control samples. Luminescence was measured every 1 min after the addition of elicitation solution for a period of 40 min using TECAN Infinite 200 PRO microplate reader and signal integration time was 0.5s. ROS measurements were expressed as means of RLU (Relative Light Units).

### Seedling Growth Inhibition

Seeds were sown on ½ MS medium supplemented with 1% agar, stratified for 2 days in the dark at 4°C, and incubated for 5 days at 22°C under a 16/8 h photoperiod. Five days old seedlings were then transferred to liquid ½ MS medium containing 1% sucrose and then left for 1 day to recover before supplementation with 1 μM flg22. The seedlings were then weighed at 10 days after treatment in three independent experiments.

### PAMP-Induced Callose Deposition

6 days old seedlings (six independent plants per treatment) grown in ½ MS medium containing 1% sucrose in a 12 well plate were treated with 1 μm of flg22 or water for 24 h. After 24 h, plants are placed in acetic acid:ethanol (1:3) over night to de-stain the chlorophyll from the leaf. Following the de-staining step plants were rehydrated with successive additions of ethanol (50% v/v) for 1 h, then 5 ml ethanol (30% v/v) for 1 h, and finally water for 2 h with two changes for washing. The plants were then incubated in the dark over night with 0.01% aniline blue in 150 mM K_2_HPO_4_. The following day individual leaves were mounted on slides in 50% glycerol, and observed *via* epifluorescence microscopy under UV illumination with a DAPI filter (Nikon). Pictures from three independent experiments was collected and the number of callose deposited calculated using the automated cell counting in ImageJ and results were given in number of cells per square pixel.

### Subcellular Localization

8-day-old roots of stable transgenic homozygous lines of *Arabidopsis* plants overexpressing 35S::*LINC1*-GFP was used. Samples were visualized for GFP expression using a confocal laser-scanning microscope (Zeiss LSM).

### Transcriptome Analysis

Total RNA was prepared from 2-week-old plants. Total RNA was extracted with NucleoSpin^®^ RNA Plant extraction kit (Macherey-Nagel) according to the manufacturer’s instructions. RNA was eluted in 60 μl RNase-free water. For RNA-seq, mRNA sequencing libraries were prepared from 1 μg of total RNA using TruSeq Stranded mRNA Library Prep Kit (Illumina). The libraries were pooled together and sequenced on a HiSeq4000 (Illumina) system. Three biological replicates were analyzed for each condition. Paired-end sequencing of RNA-Seq samples was performed using Illumina GAIIx with a read length of 100 bp. Reads were quality-controlled using FASTQC (http://www.bioinformatics.babraham.ac.uk/projects/fastqc/). Trimmomatic was used for trimming of adaptor sequences. Parameters for read quality filtering were set as follows: Minimum length of 36 bp; Mean Phred quality score greater than 30; Leading and trailing bases removal with base quality below 3; Sliding window of 4:15. TopHat v2.0.9 was used for alignment of short reads to the *A*. *thaliana* genome TAIR10, Cufflinks v2.2.0 for transcript assembly and differential expression. To identify differentially expressed genes, specific parameters (*p*-value: 0.05; statistical correction: Benjamini Hochberg; FDR: 0.05) in cuffdiff were used. Post-processing and visualization of differential expression were done using cummeRbund v2.0.0. Genes were considered as regulated if fold change was more than 1.5 and *p*-value < 0.05 compared to mock condition. Identified de-regulated genes were used to generate HCL tree using Multi Experiment Viewer (MeV 4.9.0 version, TM4,https://sourceforge.net/projects/mev-tm4/files/mev-tm4/MeV% 204.9.0/). The normalization for the raw data was carried out for every gene and hierarchical clustering was performed under Euclidian distances, average linkage and leaf order optimization. Gene enrichment analyses were performed using AgriGO (http://systemsbiology.cau.edu.cn/agriGOv2/).

### Quantitative RT-PCR

The quantitative PCR was carried out using SsoAdvanced™ Universal SYBR^®^ Green Supermix (Bio-Rad) in the CFX96 Touch™ Real-Time PCR Detection System (Bio-Rad). The relative expression values were determined using *ACTIN2* (At3g18780) and *UBIQUITIN10* (At4g05320) as reference genes and comparative analysis was carried out by Bio-Rad CFX manager software with the Ct method (2-ΔΔCt). Normalized gene expression was expressed relative to wild-type controls in each experiment.

### Phytohormone Measurements

For phytohormones quantification, the leaves from 4-weeks old (wild-type, *linc1-1* and OE) plants were collected after inoculated as described above. 24 h after treatment, the plants were harvested, immediately frozen in liquid nitrogen, freeze-dried overnight in a Liophylazer, and grinded in a Gino grinder (for 2 cycles of 45 s each, at 1150 rpm). They were then aliquoted in 1.5 ml tubes (around 10 mg) and extracted with 1.0 ml of 70% methanol containing the respective phytohormones internal standards (d6-JA and d5-SA). The measurements were performed on an Agilent 1100 HPLC system (Agilent Technologies, Böblingen, Germany) connected to a LTQ Iontrap mass spectrometer (Thermo Scientific, Bremen, Germany), and the quantification was based on a calibration curve using original SA, JA, and JA-Ile standards (Almeida Trapp et al., 2014).

### MeJA-Mediated Inhibition of Root Growth

Seeds were plated on ½ MS agar media plates with or without 50 μM MeJA (Sigma-Aldrich), placed for 2 days at 4°C in the dark, and subsequently placed vertically for 10 days. Three independent biological replicates were measured for each sample, and similar results were obtained from each independent experiment.

### Statistical Analysis

Statistical significance based on two-way ANOVA analysis was performed with Prism 7 (GraphPad Software).

## Data Availability Statement

The RNAseq data discussed in this publication have been deposited in NCBI’s Gene Expression Omnibus and are accessible through GEO Series accession number GSE118854(https://www.ncbi.nlm.nih.gov/geo/query/acc.cgi?acc=GSE118854).

## Author Contributions

MJ, NR, and HH designed the study. MJ, MA-T, MM, and NR performed experimental work. MJ, KM, MA-T, MM, AM and NR analyzed data. MJ, NR and HH wrote the paper. All authors read and approved the manuscript.

## Funding

This work was supported by the King Abdullah University of Science and Technology grant (BAS/1/1062-01-01 and URF/1/2965-01-01 to H.H.).

## Conflict of Interest

The authors declare that the research was conducted in the absence of any commercial or financial relationships that could be construed as a potential conflict of interest.
