# Supplementary material for: The Lamin-Like LITTLE NUCLEI 1 (LINC1) Regulates Pattern-Triggered Immunity and Jasmonic Acid Signaling
Source: Front Plant Sci. 2020 Jan 9;10:1639. doi: 10.3389/fpls.2019.01639 (PMC6963418; doi:10.3389/fpls.2019.01639)
Supplement: Supplementary file 1 [file DataSheet_1.pdf]

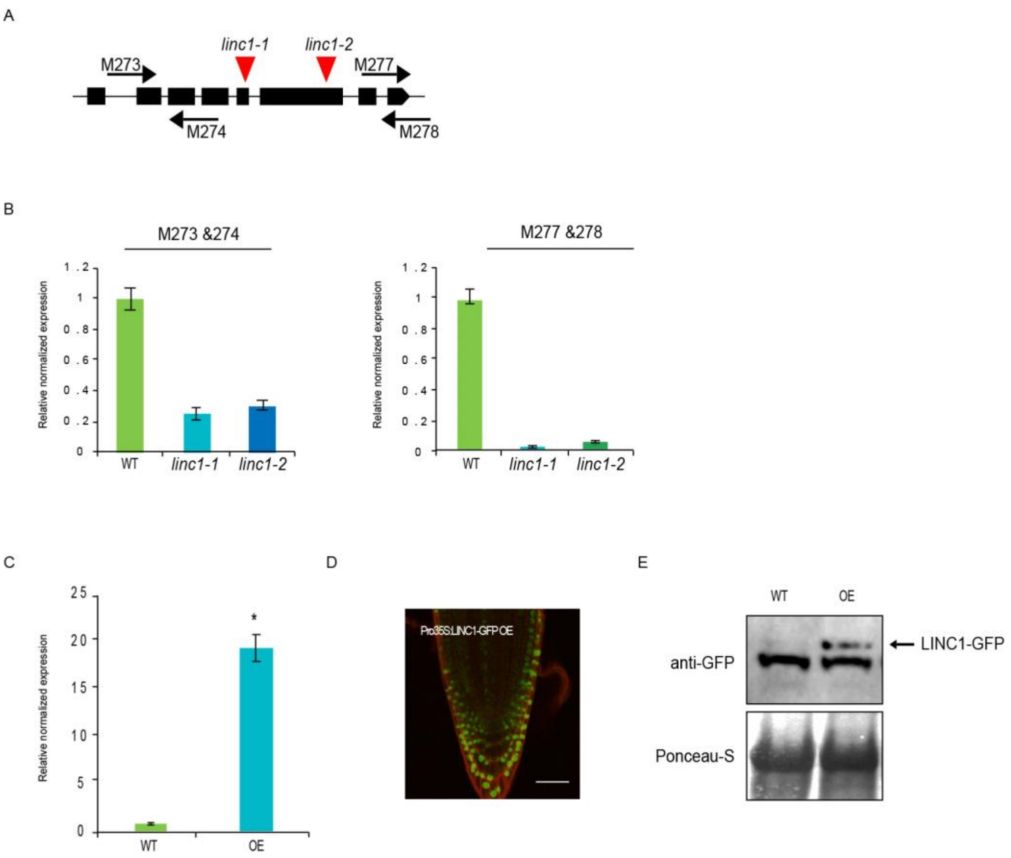

Figure S1: Genomic organization and T-DNA insertional alleles of LINC1. A. Genomic organization of LINC1: exons are shown as solid black bars and introns as the thinner line. Transcription proceeds from left to right. The positions of T- DNA insertions from the SALK SIGnAL collection (Alonso et al., 2003) are depicted as triangles. The arrows denote primers for qRT-PCR. B. qRT-PCR of homozygous linc1-1 and linc1-2 mutants demonstrating that transcription downstream of the insertion site is abolished. Gene expression was normalized to internal control UBQ10. C. The transcript levels of LINC1 in overexpression line (Pro35S:LINC1- GFP). The relative transcript levels of LINC1 were monitored by qRT-PCR. Gene expression was normalized to internal control UBQ10. Data are represented as mean ± SEM (n=3 biological replicates). D. LINC1 expression and protein localization in Arabidopsis roots (10 days old seedlings). Stable transgenic plants overexpressing LINC1–GFP (OE) reveal that LINC1 is mainly localized to the nucleus. Confocal laser scanning microscopy images of GFP fluorescence (green) and propidium iodide (PI) fluorescence (red) of Arabidopsis seedlings in the root apical meristem. Scale bar: 20 µm. E. Immunoblot of WT, OE plant expressing 35S::LINC1–GFP probed with anti-GFP antibody (upper panel) and protein loading control with Ponceau S staining (lower panel).

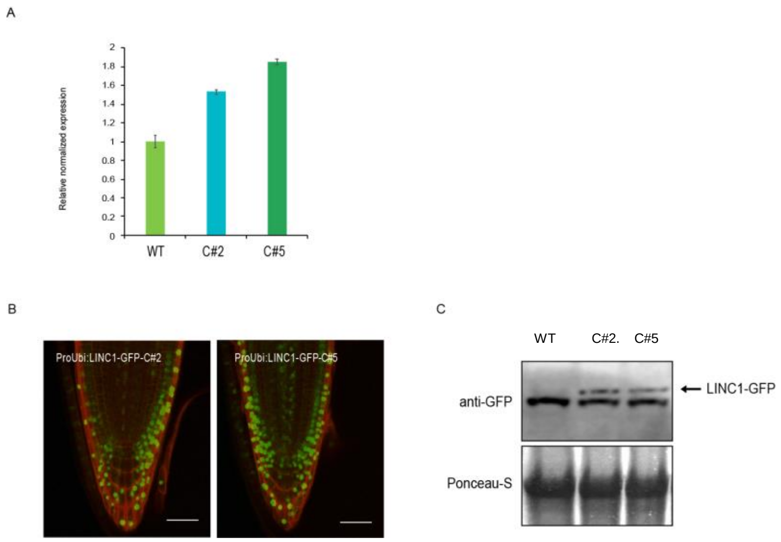

Figure S2: LINC1 complementation lines. A. The transcript levels of LINC1 in complemented lines. The relative transcript levels of LINC1 were monitored by qRT-PCR. Gene expression was normalized to internal control UBQ10 and Actin. B. LINC1 expression and protein localization in Arabidopsis roots (10 days old seedlings). Stable transgenic complementation plants (C#2 and C#5) expressing LINC1–GFP under Ubi promoter reveal that LINC1 is mainly localized to the nucleus. Confocal laser scanning microscopy images of GFP fluorescence (green) and propidium iodide (PI) fluorescence (red) of Arabidopsis seedlings in the root apical meristem. Scale bar: 20 µm. E. Immunoblot of WT, complementation lines expressing Ubi::LINC1–GFP probed with anti-GFP antibody (upper panel) and protein loading control with Ponceau S staining (lower panel).

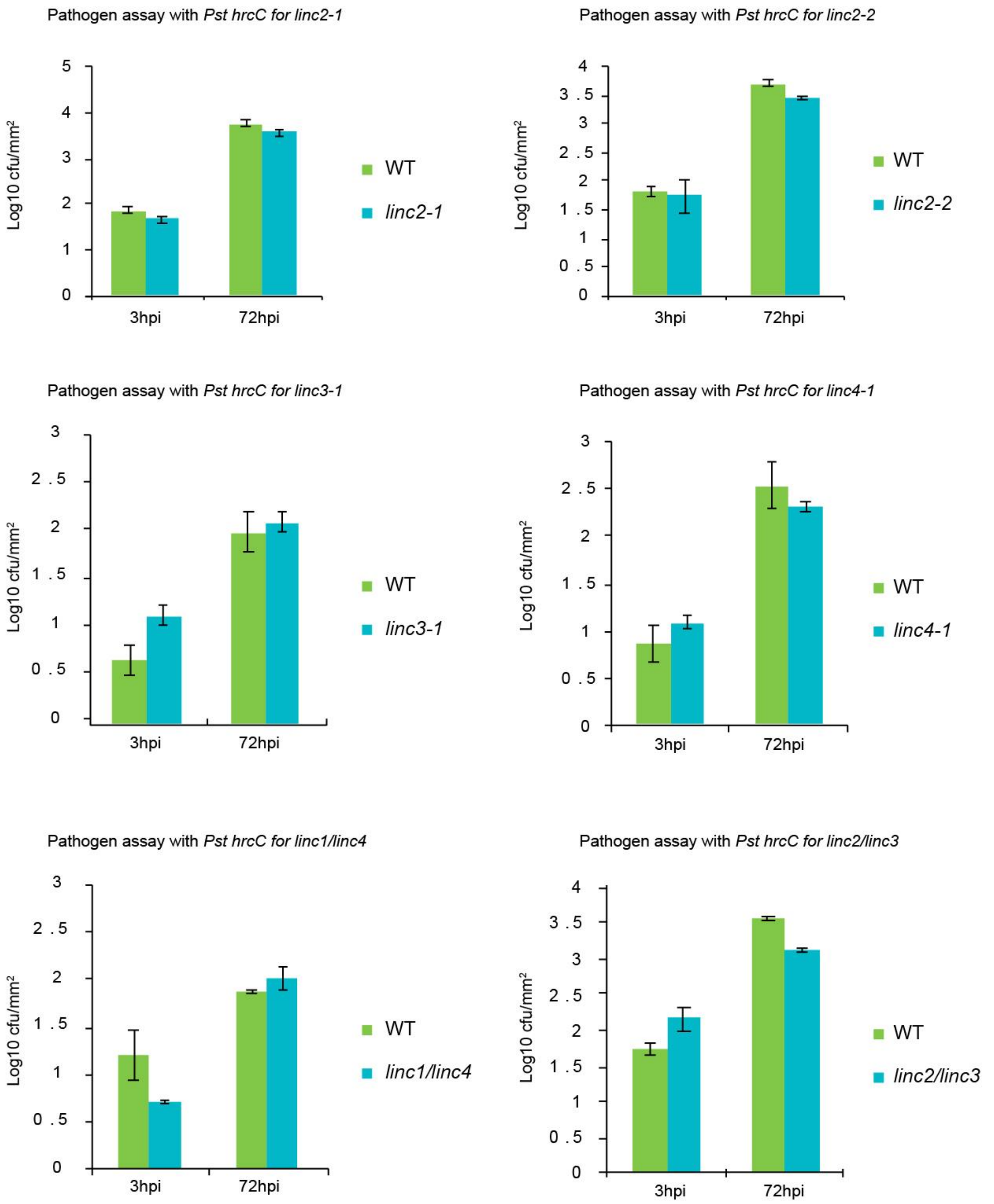

Figure S3: *Pst hrcC-* infection phenotype of LINC1 mutants. Four-week-old wild-type, *linc2-1, linc2-2, linc3-1, linc4-1, linc1-1 linc4- 1* and *linc2-1 linc3-1* plants were spray inoculated with bacterial *Pst hrcC-* suspension at an OD600=0.2, and bacteria inside leaf tissue was quantified at 3 and 72 hpi. Statistical significance was analyzed by two-way anova, asterisks indicate significant differences compared to Wild Type - * p 0.05, ** p 0.01, *** p 0.001.

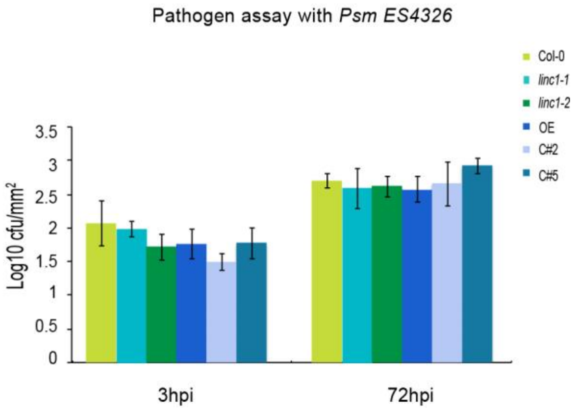

Figure S4: *Psm ES4326* infection phenotype of LINC1 plants. Four- week-old wild-type, *linc1-1*, *linc1-2*, OE, C#2 and C#5 plants were spray inoculated with bacterial *Psm ES4326* suspension at an OD600=0.002, and bacteria inside leaf tissue was quantified at 3 and 72 hpi. Statistical significance was analyzed by two-way anova, asterisks indicate significant differences compared to Wild Type, * p≤0.05, ** p ≤ 0.01, *** p ≤ 0.001.

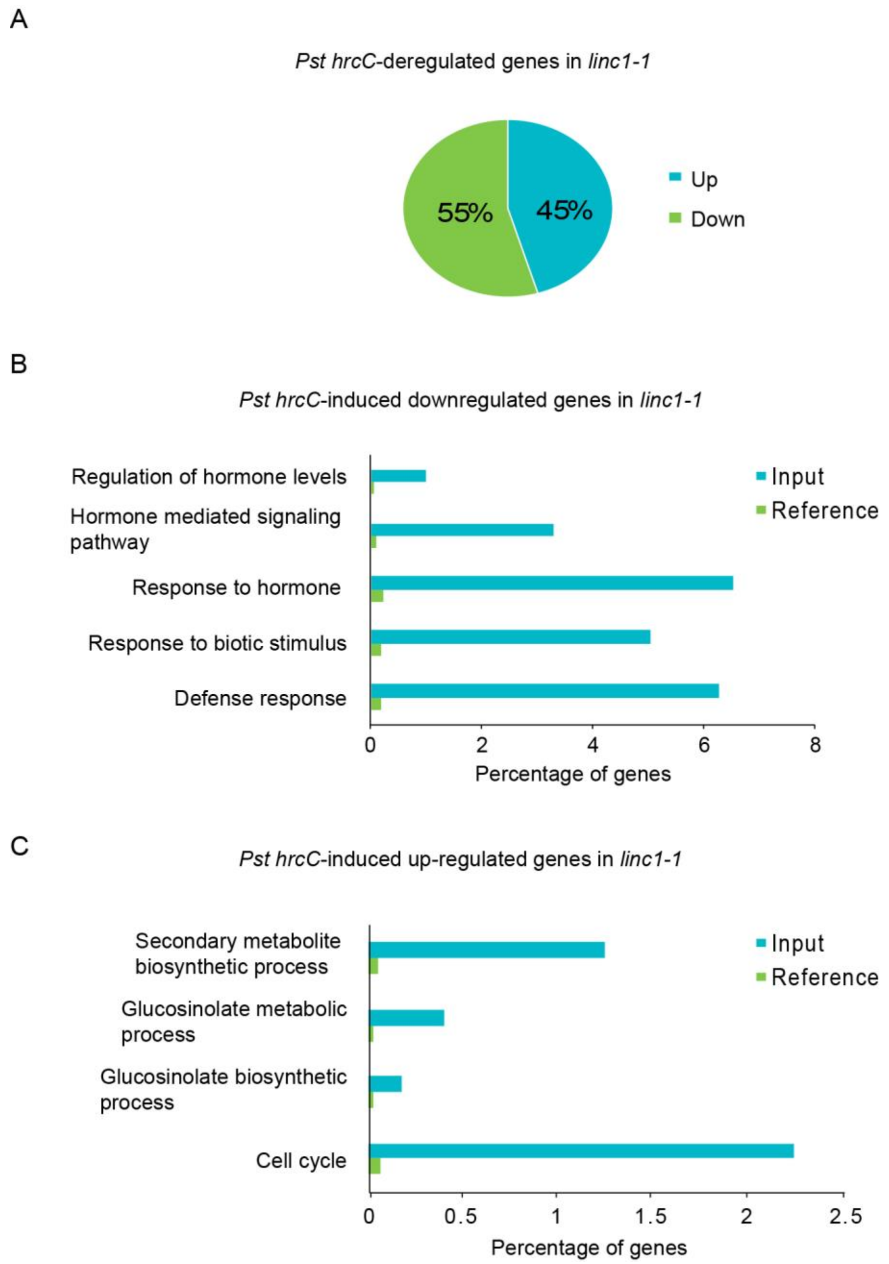

Figure S5: Differentially expressed genes in *linc1-1* upon *Pst hrcC-* infection. A. *Pst hrcC-*induced DEGs in *linc1-1*. 55 % of *linc1-1* DEGs are downregulated (green, 585 genes) whereas 45 % are upregulated (red, 385 genes). B. Enrichment of genes with GO terms related to defense response for cluster 9 and cluster 13. The fold enrichment was calculated based on the frequency of genes annotated to the term compared with their frequency in the genome. C. Enrichment of genes with GO terms related to cell cycle and GS metabolic and biosynthetic processes for cluster 7 and 14. The fold enrichment was calculated based on the frequency of genes annotated to the term compared with their frequency in the genome.

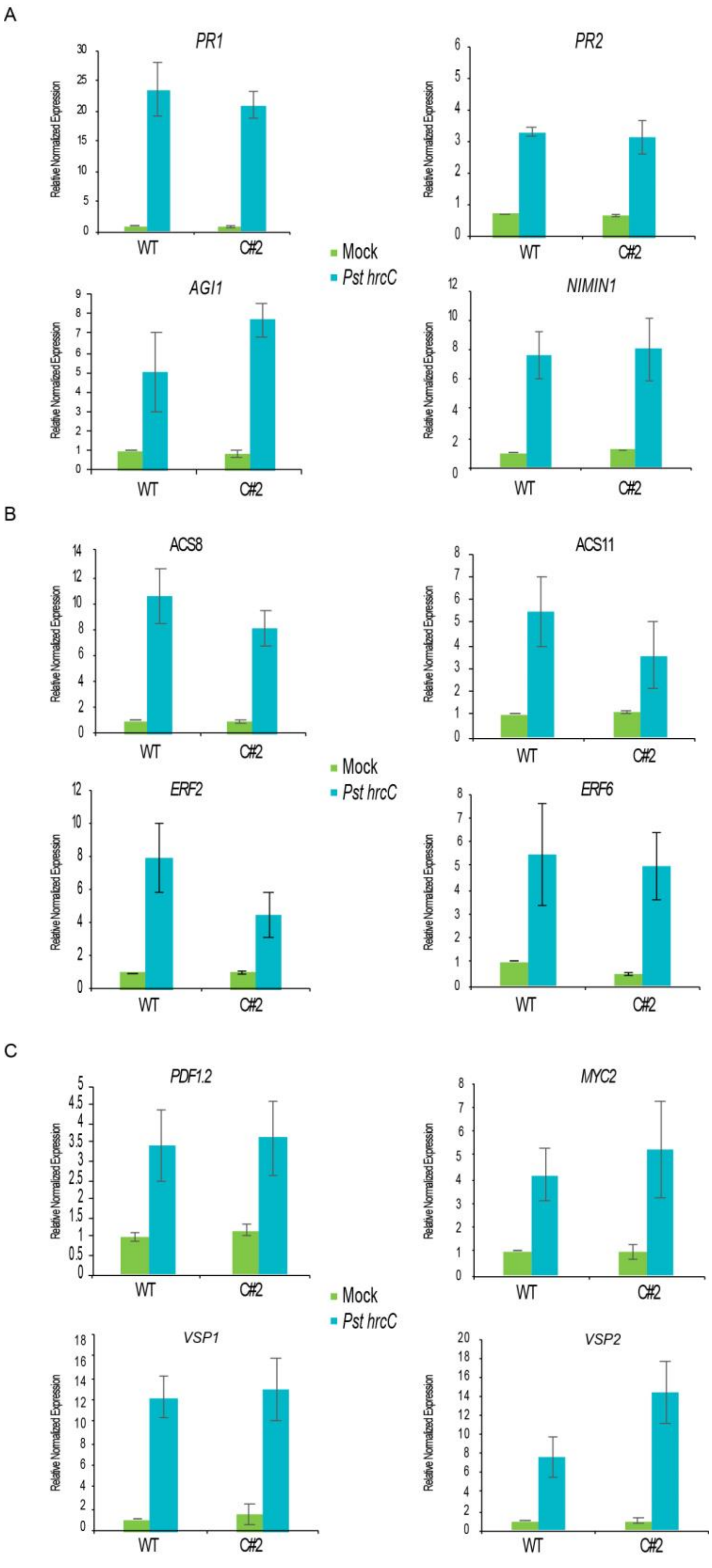

Figure S6: Recovery of wild-type expression levels in the C#2 line. A. qRT-PCR analysis of SA-related genes regulated by *LINC1*. B. qRT-PCR analysis of ET-related genes regulated by *LINC1*. C. qRT-PCR analysis of JA-related genes regulated by *LINC1*. Gene expression was normalized to internal control *UBQ10* and actin. The data shown are means from three biological replicates. Statistical significance was analyzed by two-way anova, asterisks indicate significant differences compared to Wild Type, * p≤0.05, ** p ≤ 0.01, *** p ≤ 0.001.

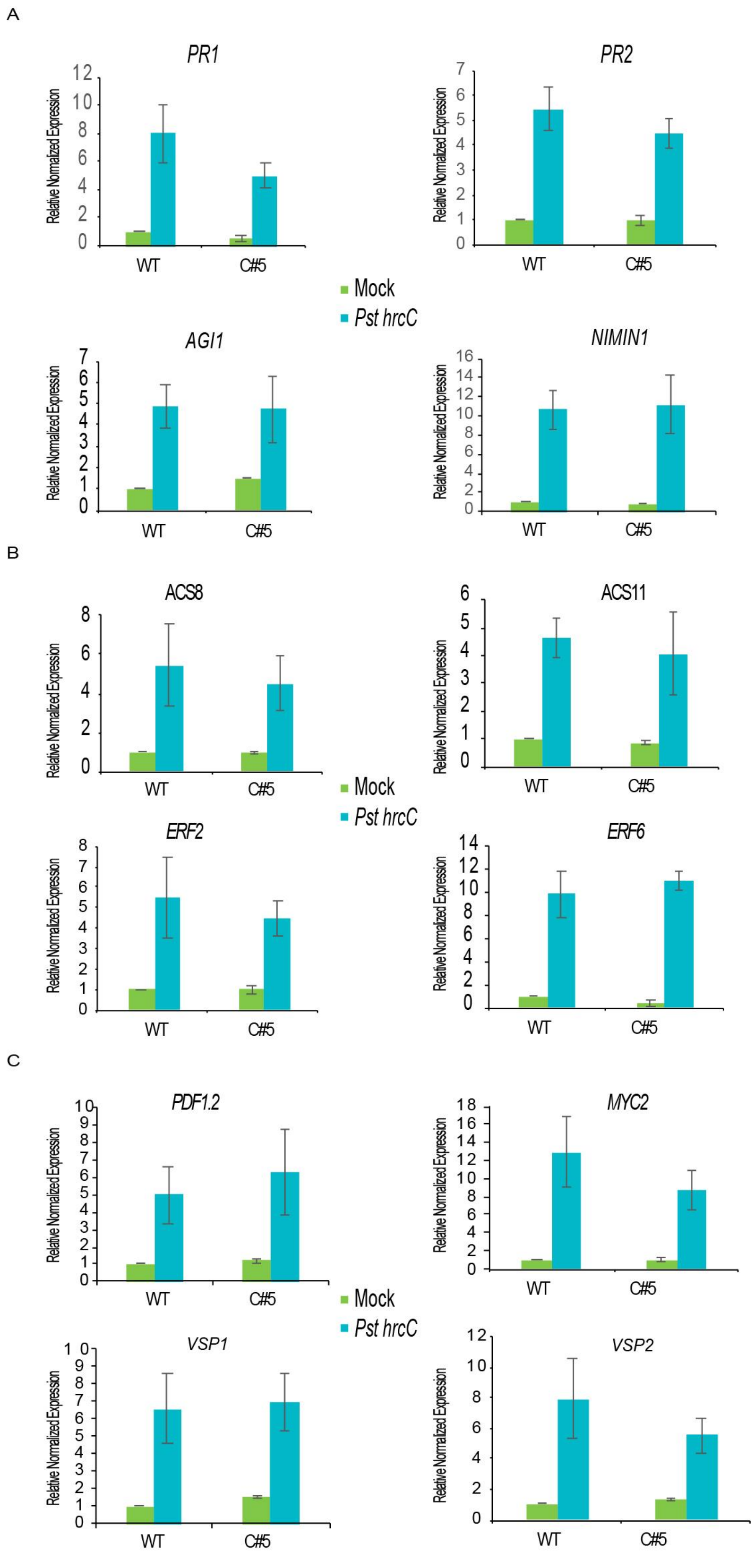

Figure S7: Recovery of wild-type expression levels in the C#5 complementation line. A. qRT-PCR analysis of SA-related genes regulated by *LINC1*. B. qRT-PCR analysis of ET-related genes regulated by *LINC1*. C. qRT-PCR analysis of JA-related genes regulated by *LINC1*. Gene expression was normalized to internal control *UBQ10* and actin. The data shown are means from three biological replicates. Statistical significance was analyzed by two-way anova, asterisks indicate significant differences compared to Wild Type, * p≤0.05, ** p ≤ 0.01, *** p ≤ 0.001.

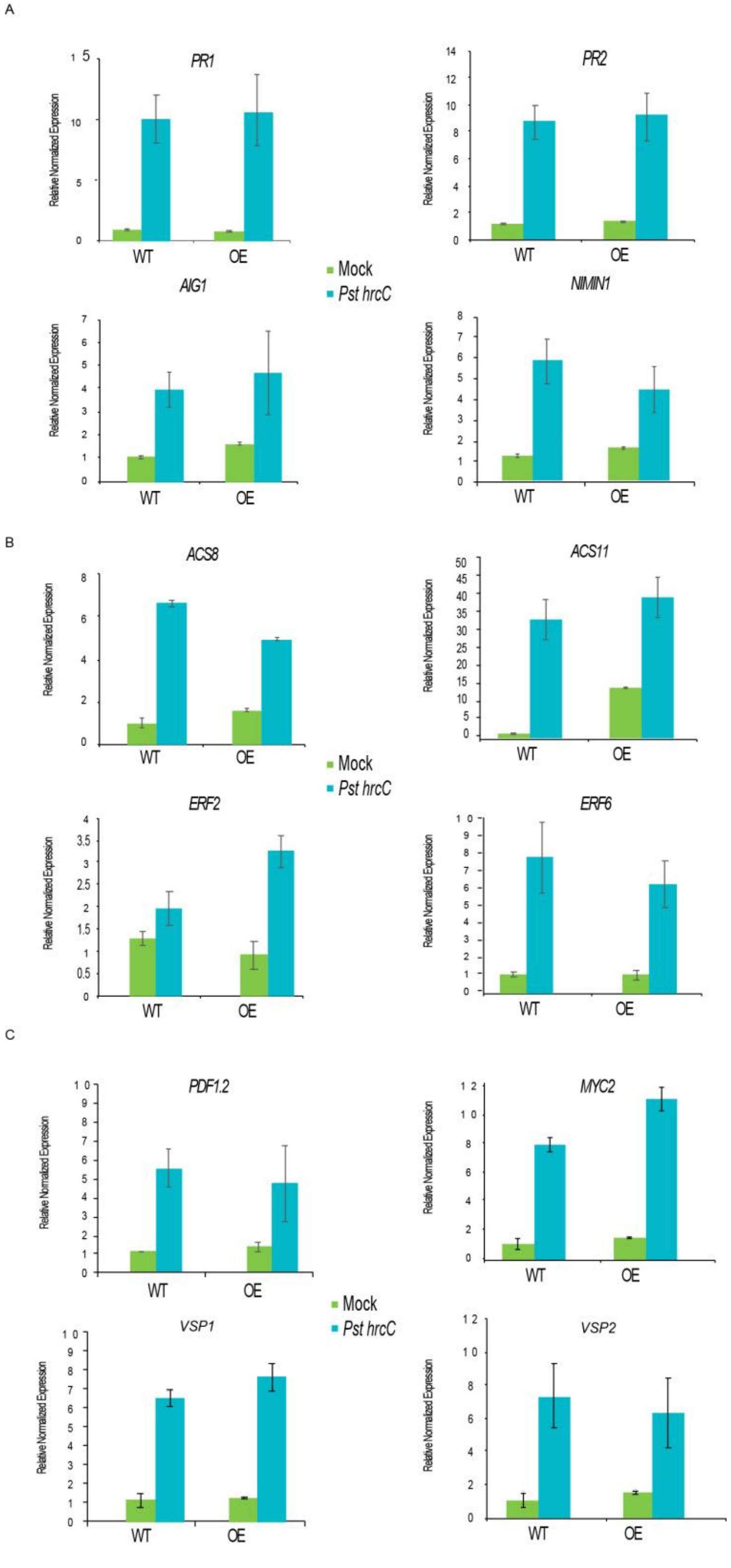

Figure S8: Recovery of wild-type expression levels in the LINC1 OE line. A. qRT-PCR analysis of SA-related genes regulated by *LINC1*. B. qRT-PCR analysis of ET-related genes regulated by *LINC1*. C. qRT-PCR analysis of JA-related genes regulated by *LINC1*. Gene expression was normalized to internal control *UBQ10* and actin. The data shown are means from three biological replicates. Statistical significance was analyzed by two-way anova, asterisks indicate significant differences compared to Wild Type, * $p \leq 0.05$, ** $p \leq 0.01$, *** $p \leq 0.001$.

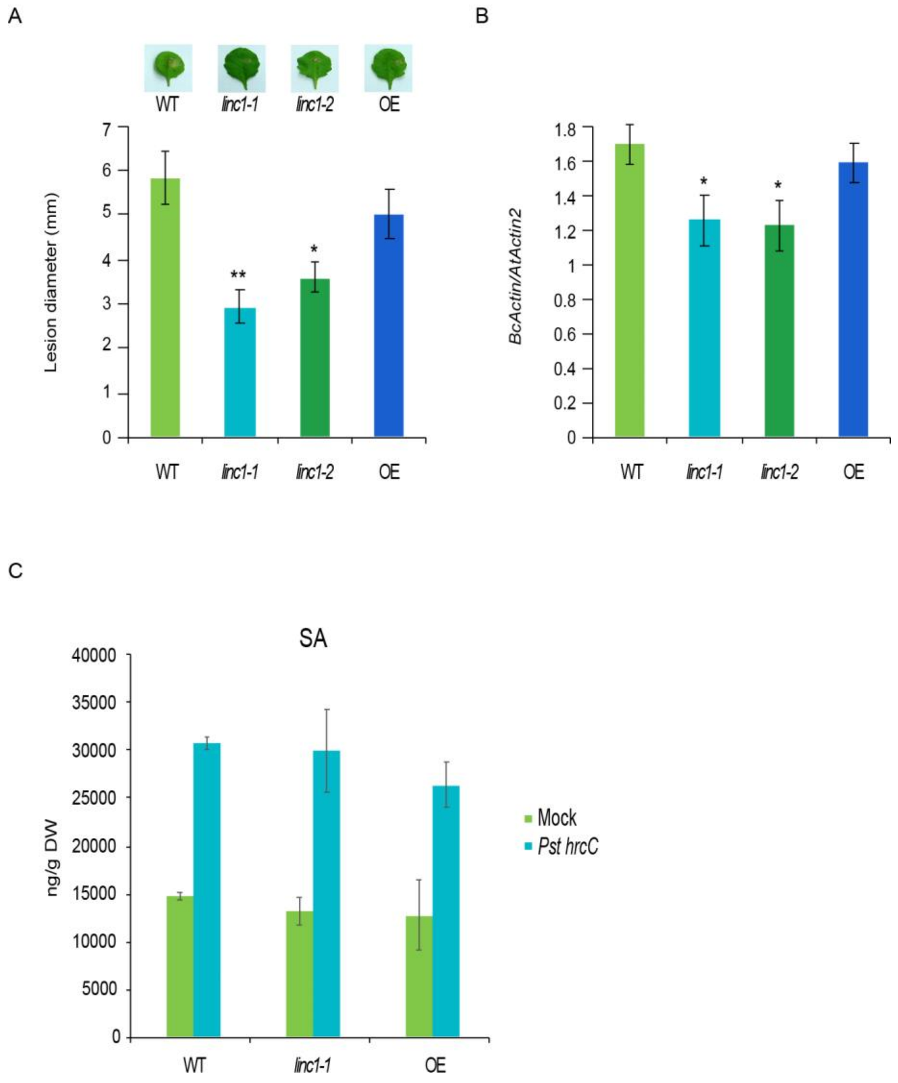

Figure S9: Phenotype of *LINC1* lines upon *Botrytis cinerea* infection. A. Four-week-old wild-type, *linc1-1, linc1-2* and OE plants were drop inoculated with a *B. cinerea* suspension at a density of $5*10^6$ spores/ml. The fungal infection was quantified after 72 hpi by measuring the lesion diameter. B. qPCR analysis of B. cinerea infection quantified by measuring the transcript levels of *B. cinerea* actin gene (*BcActin*) and the *Arabidopsis* actin gene (*AtActin2*) was used as an internal control C. SA levels in wild-type, *linc1-1* and OE plants. Leaves of 3-4 week-old wild-type and *linc1-1* mutant were infiltrated with Pst hrcC- (OD600=0.2). Leaves were collected at 0 and 1 dpi, and SA was extracted and analyzed. The data shown are means from three biological replicates. Statistical significance was analyzed by two-way anova, asterisks indicate significant differences compared to Wild Type, * $p \leq 0.05$, ** $p \leq 0.01$, *** $p \leq 0.001$.

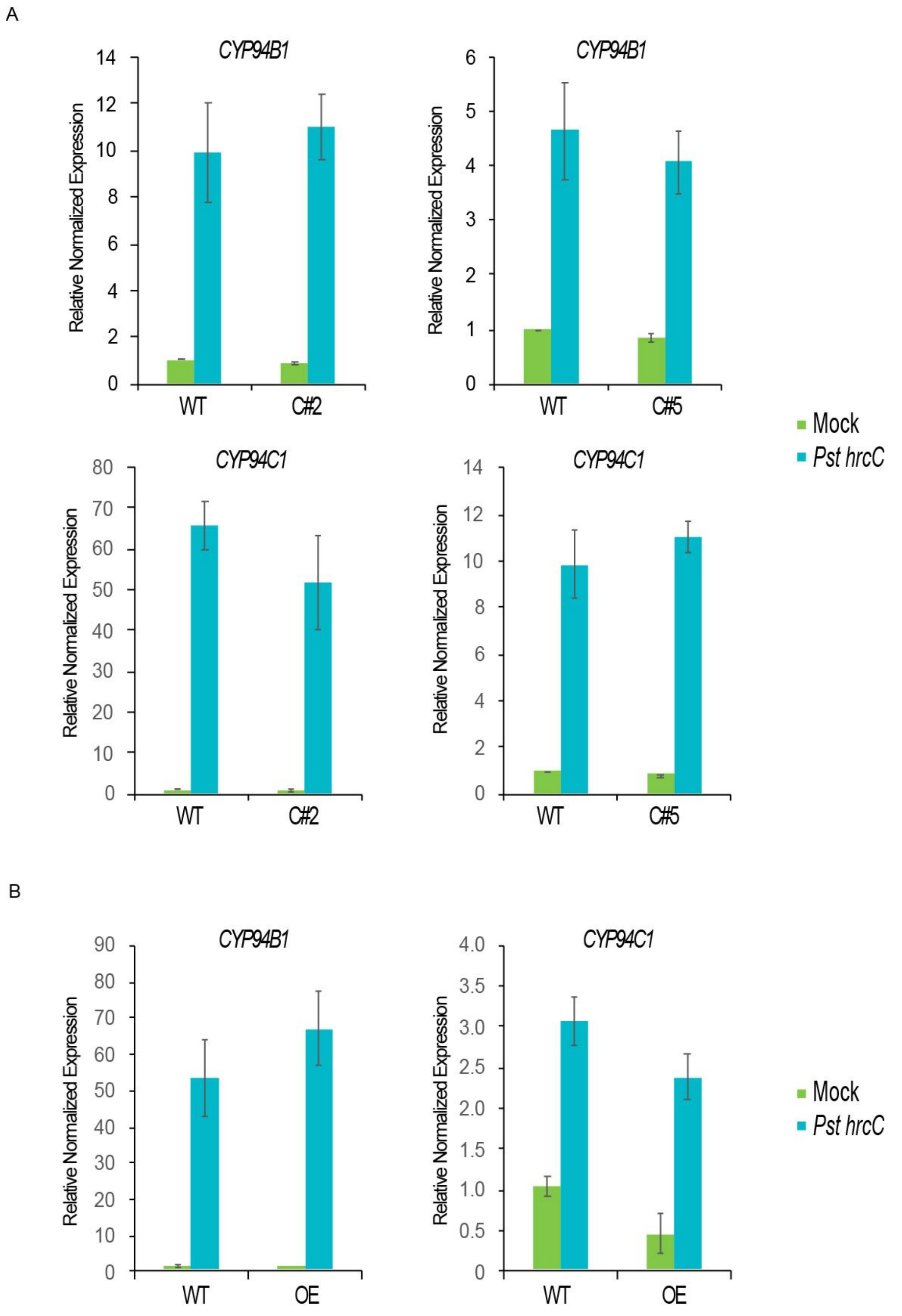

Figure S10: Gene expression levels in the C#2, C#5 and OE lines for *CYP94B1* and *CYP94C1*. A. qRT-PCR analysis of *CYP94B1* and *CYP94C1* genes in the C#2 and C#5 lines. B. qRT-PCR analysis of *CYP94B1* and *CYP94C1* genes in the LINC1 OE line. Gene expression was normalized to internal control *UBQ10* and actin. The data shown are means from three biological replicates. Statistical significance was analyzed by two-way anova, asterisks indicate significant differences compared to Wild Type, * $p \leq 0.05$, ** $p \leq 0.01$, *** $p \leq 0.001$.

A

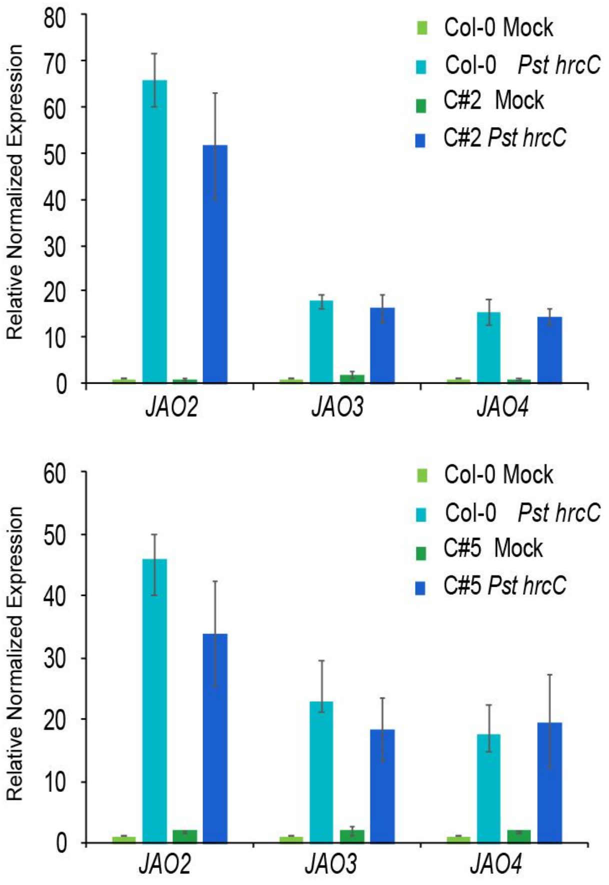

B

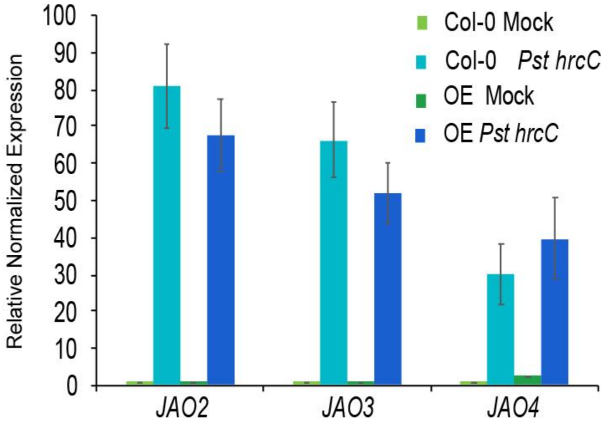

Figure S11: Gene expression levels of *JAO2, JAO3* and *JAO4* in C#2, C#5 and OE lines. A. qRT-PCR analysis of *JAO2, JAO3* and *JAO4* genes in the C#2 and C#5 lines. B. qRT-PCR analysis of *JAO2, JAO3* and *JAO4* genes in the OE line. Gene expression was normalized to internal control *UBQ10* and actin. The data shown are means from three biological replicates. Statistical significance was analyzed by two-way anova, asterisks indicate significant differences compared to Wild Type, * p≤0.05, ** p ≤ 0.01, *** p ≤ 0.001.

A

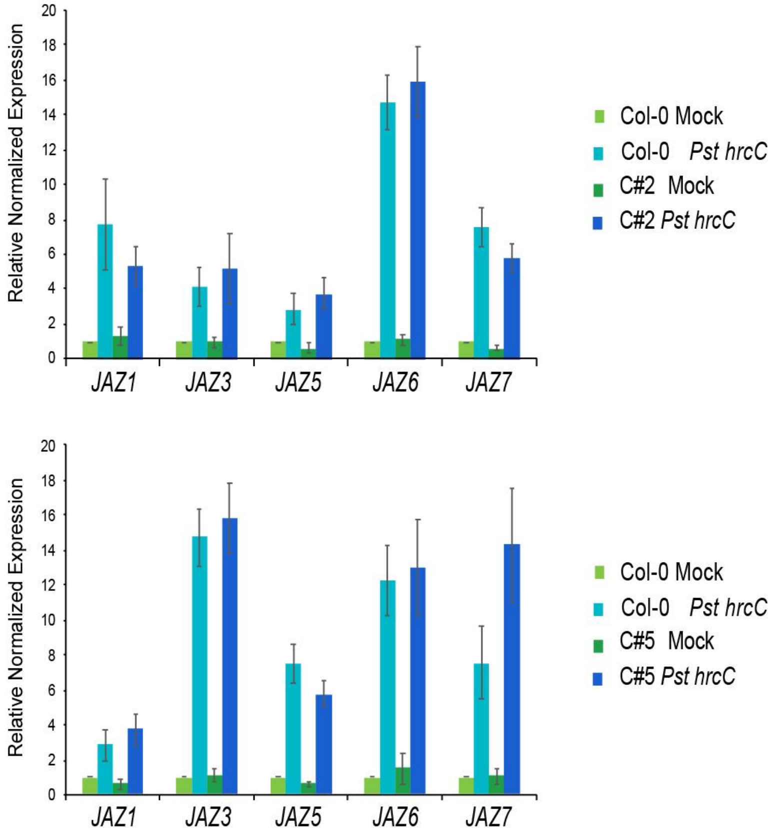

B

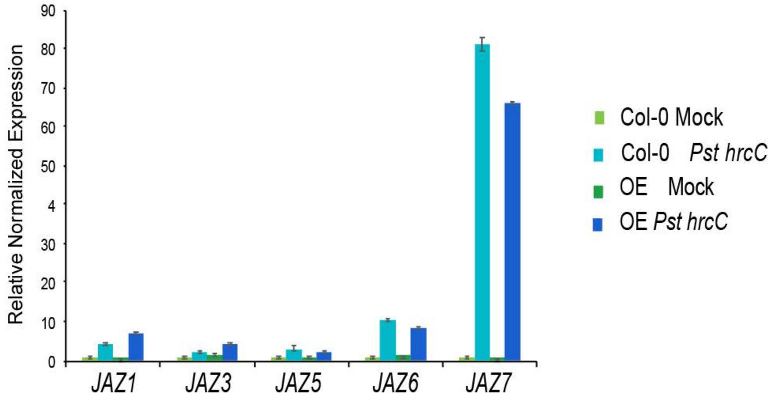

Figure S12: Gene expression levels of JAZ1, 3, 5, 6 and 7 in C#2, C#5, and OE lines. A. qRT-PCR analysis of *JAZ1, 3, 5, 6* and *7* genes in the C#2 and C#5 lines. B. qRT-PCR analysis of *JAZ1, 3, 5, 6* and *7* genes in the OE line. Gene expression was normalized to internal control *UBQ10* and actin. The data shown are means from three biological replicates. Statistical significance was analyzed by two-way anova, asterisks indicate significant differences compared to Wild Type, * $p \leq 0.05$, ** $p \leq 0.01$, *** $p \leq 0.001$.

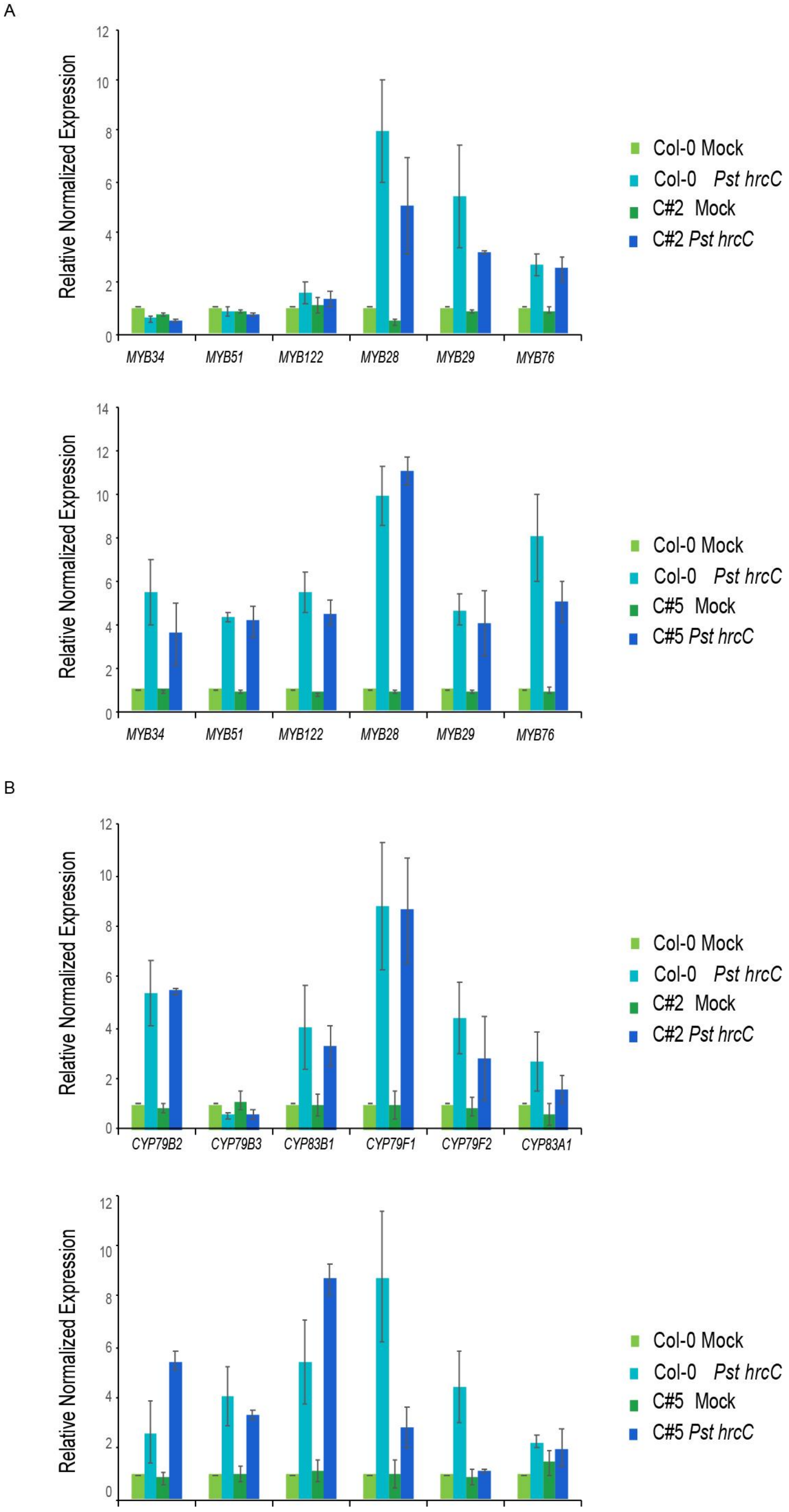

Figure S13: Gene expression levels for GS-related genes in C#2 and C#5 lines. A. qRT-PCR analysis of expression levels of transcription factor genes involved in the biosynthetic pathway of indolic and aliphatic glucosinolates in the C#2 and C#5 lines. B. qRT-PCR analysis of expression levels of biosynthetic genes involved in the biosynthetic pathway of indolic and aliphatic glucosinolates in the C#2 and C#5 lines. Gene expression was normalized to internal control *UBQ10* and actin. The data shown are means from three biological replicates. Statistical significance was analyzed by two-way anova, asterisks indicate significant differences compared to Wild Type, * p≤0.05, ** p ≤ 0.01, *** p ≤ 0.001.

A

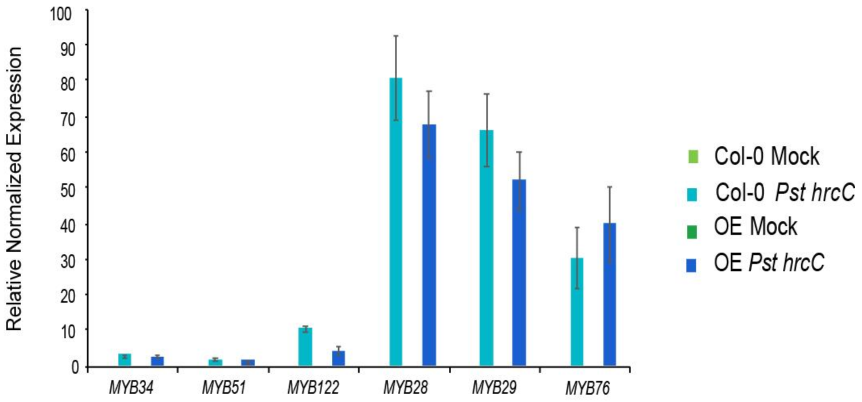

B

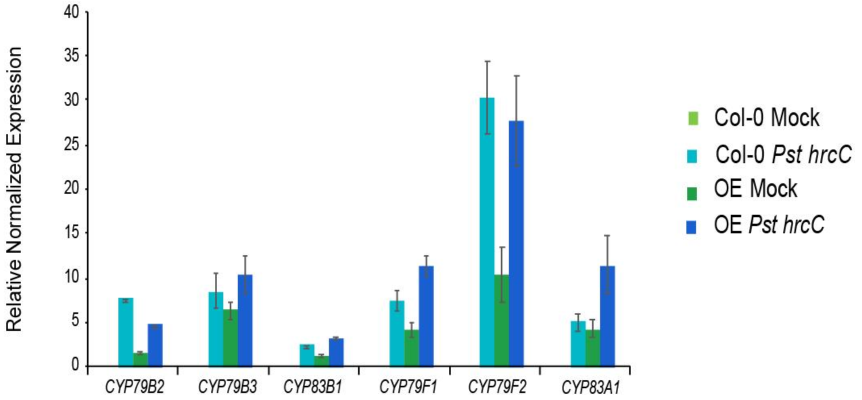

Figure S14: Gene expression levels for GS-related genes in the OE line. A. qRT-PCR analysis of expression levels of transcription factor genes involved in the biosynthetic pathway of indolic and aliphatic glucosinolates in the OE line. B. qRT-PCR analysis of expression levels of biosynthetic genes involved in the biosynthetic pathway of indolic and aliphatic glucosinolates in the OE line. Gene expression was normalized to internal control *UBQ10* and actin. The data shown are means from three biological replicates. Statistical significance was analyzed by two-way anova, asterisks indicate significant differences compared to Wild Type, * $p \leq 0.05$, ** $p \leq 0.01$, *** $p \leq 0.001$.
